# I-KID study protocol: evaluation of efficacy, outcomes and safety of a new infant haemodialysis and ultrafiltration machine in clinical use: a randomised clinical investigation using a cluster stepped-wedge design

**DOI:** 10.1136/bmjpo-2021-001224

**Published:** 2021-10-18

**Authors:** Heather J Lambert, Shriya Sharma, John N S Matthews, the I-KID Protocol Development Group

**Affiliations:** 1 Paediatric Nephrology, Great North Children's Hospital, Newcastle Upon Tyne, UK; 2 Department of Child Health, Newcastle University, Newcastle upon Tyne, UK; 3 Faculty of Medical Sciences, Newcastle University, Newcastle upon Tyne, Tyne and Wear, UK; 4 Department of Mathematics, Statistics & Physics and Population Health Sciences Institute, Newcastle University, Newcastle upon Tyne, Tyne and Wear, UK

**Keywords:** nephrology, neonatology, technology

## Abstract

**Introduction:**

The I-KID study aims to determine the clinical efficacy, outcomes and safety of a novel non-CE-marked infant haemodialysis machine, the Newcastle Infant Dialysis Ultrafiltration System (NIDUS), compared with currently available therapy in the UK. NIDUS is specifically designed for renal replacement therapy in small babies between 0.8 and 8 kg.

**Methods and analysis:**

The clinical investigation is taking place in six UK centres. This is a randomised clinical investigation using a cluster stepped-wedge design. The study aims to recruit 95 babies requiring renal replacement therapy in paediatric intensive care units over 20 months.

**Ethics and dissemination:**

The study has high parent and public involvement at all stages in its design and parents will be involved in dissemination of results to parents and professionals via publications, conference proceedings and newsletters. The study has has ethics permissions from Tyne and Wear South Research Ethics Committee.

**Trial registration numbers:**

IRAS ID number: 170 481

MHRA Reference: CI/2017/0066

ISRCT Number: 13 787 486

CPMS ID number: 36 558

NHS REC reference: 16/NE/0008

Eudamed number: CIV-GB-18-02-023105

Link to full protocol v6.0: https://fundingawards.nihr.ac.uk/award/14/23/26

**What is already known on this topic??**

- Babies in paediatric intensive care unit may develop acute renal failure and require therapeutic support.
- Renal replacement options for babies are limited because of their small size and limitations of technology.
- Current haemodialysis and filtration systems in use in UK are not recommended or licensed for children under 8 kg.

**What this study hopes to add?**

- Renal replacement methods for infants under 8 kg in paediatric intensive care unit will be compared.
- The efficacy, outcomes and safety of a new infant haemodialysis device will be assessed.
- Usability of the new device in normal clinical settings outside of the development centre will be examined.

## Introduction

Young babies requiring renal replacement therapy (RRT) present specific therapeutic challenges because of their small size and the current technology available. Publications indicate similar problems faced by clinicians worldwide who use adult devices because of lack of alternatives, and the need for solutions including improved device technology.^1 2^

This clinical investigation protocol is designed to determine the clinical efficacy, outcomes and safety of a novel non-CE-marked infant haemodialysis (HD) machine, the Newcastle Infant Dialysis Ultrafiltration System (NIDUS), compared with currently available RRT in the UK. NIDUS is designed for use in babies between 0.8 and 8 kg. There is evidence from a previous single-centre pilot study to anticipate NIDUS has the potential to contribute significant benefits to small babies needing RRT.^3^

The proposed clinical investigation is a result of a multicentre collaboration between clinicians, scientists, academics, with significant parent and public involvement, throughout its development; working with a manufacturing company, Allmed. The results will have potential to change clinical practice.

The NIDUS machine uses a smaller circuit volume than current devices. Pilot data from the development centre have suggested management of fluid overload and renal failure is possible for small infants, with the potential for reduced exposure to blood products, and more precise control of ultrafiltration (UF) and dialysis.^3^ Nurses have reported ease of use of the NIDUS within the design centre but this requires evaluation in standard clinical environments.

### Background

There are several populations of babies requiring RRT. Those included in this study are unwell infants in paediatric intensive care units (PICU), who mostly do not have intrinsic renal disease and therefore have good potential for renal recovery. Many are postoperative, especially postcardiac surgery, whose major problem is an acute kidney insult, fluid overload and poor urine output, and others who are septic or have renal failure as part of multiorgan failure. Although mortality and morbidity in PICU varies and is related to the underlying diagnosis, survival of babies in PICU is worse in those with fluid overload^4^ or needing RRT,^5^ of whom up to 20%–40% may die.^5–9^ RRT is supportive until kidney recovery and although most survivors are independent of RRT at discharge from PICU, data on chronic renal sequelae are lacking. Children requiring RRT in PICU have been reported to have longer length of stay and required more days of ventilator support.^8^ There are over 200 infants per year in the UK receiving treatment with continuous RRT (CRRT) in PICU.^10 11^

Some babies will be excluded—for example, those with an inborn error of metabolism such as urea cycle defects causing hyperammonaemia, as they require emergency, very rapid removal of toxic metabolites by higher than normal dialysis clearances,^12^ and babies with severe intrinsic renal disease, which is often congenital, who are usually treated with chronic peritoneal dialysis (PD) at home.

### Current RRT

PD is used frequently to support infants after open heart surgery.^5 13^ PD is technically simpler than HD; there is no lower size limit but complications are common in the smallest patients.^4^ UF is unpredictable, and chemical clearance less efficient, especially in unstable babies who develop splanchnic vasoconstriction and who also risk developing necrotising enterocolitis. This renders PD impossible, as does abdominal surgery and congenital abdominal wall defects. Larger critically ill infants with multiorgan failure are often treated with a variety of continuously delivered HD modalities (CRRT).^4 5 9^ Vascular access for HD modalities including continuous veno-venous haemofiltration (CVVH) is problematic as the size of central venous line required for adequate blood flow is disproportionately large for the size of the baby especially when a double lumen line is needed.

While there are no randomised controlled trials in infants, publications indicate recurring themes of difficulties with vascular access and blood flows, fluid balance, rapid clotting, loss of circuits and hypotensive episodes at initiation.^6–9^

Conventional HD and CRRT machines in the UK are used in PICU unlicensed as they are CE marked for use in adults and bigger children. Manufacturers quote fluid balance control as ±30 mL/hour,^14^ and they, therefore, are not licensed for babies weighing <8 kg (or approved for use in children of <20 kg in the USA). The recommended minimum 7-French, dual-lumen vascular access lines and continuous 40 mL/min blood flows are difficult to achieve in the smallest babies. Their relatively large circuit volume (50–70 mL) produces sudden dilution of blood on commencing treatment if crystalloid primed, and increases the risk of anaemia with circuit loss. Hypotension on connection is a reported problem.^6 15 16^ Blood priming risks exposing the baby abruptly to aberrant chemical and pH changes, which are reduced by predialysing the circuit.^17^ Exposure to blood transfusions increases the risk of developing tissue-type sensitisation which may be important if renal function does not recover and renal or other solid organ transplant is considered in the future.

There is one CE marked new device for smaller children, the CARPEDIEM, which is not yet available in the UK to enable comparisons,^18 19^ others, notably in USA, have adapted other adult devices like Aquadex.^9 20^

### NIDUS technology

In 1995, a group in Newcastle designed a novel HD circuit, which operated by different principles that is, by syringes, and uncoupled the baby’s blood flow capacity from the requirements of the dialysis filter.^21^ In 2005, they reported the results of automating this as a miniaturised machine (circuit volume less than 10 mL), with which four babies weighing under 4 kg were treated, using a single-lumen access line, and without the need for blood priming.^22^ This device was subsequently developed into NIDUS,^3^ which is used as the intervention device in the I-KID study. This clinical investigation will contribute to the current knowledge base and further understanding of the effects of RRT and address the need for improved technology to provide RRT effectively and safely for small babies.^1 23 24^

Safety monitoring is an important focus of this study. The NIDUS makes a downloadable constant recording of all activity data including volumes, flows, pressures, alarms and response to alarms so any alarm or event, however small, can be subsequently analysed. The NIDUS potentially provides a safer way of performing RRT on babies by using a novel circuit that allows precise ultrafiltrate control thus reducing the potential for errors in ultrafiltrate removal that would be trivial for larger children but are not for a baby. Its small circuit volume (<10 mL) potentially avoids the need for blood priming with stored blood which has associated immediate risks and long-term risks of developing sensitising antibodies.

## Methods and analysis

The study aims to evaluate the efficacy and precision of NIDUS in UF fluid removal and monitor adverse effects of RRT including use of blood product transfusion (table 1). It will also generate a safety profile in the application of NIDUS in the clinical environment.

**Table 1:** I-KID study summary

Design	A multicentre, randomised clinical investigation using a cluster stepped-wedge design
Study interventions	Control: current renal replacement therapy (either peritoneal dialysis or continuous veno-venous haemofiltration)
	Experimental intervention: renal replacement therapy using the newcastle infant dialysis ultrafiltration system
Objectives	Primary: To compare the use of a novel haemodialysis device with conventional renal replacement therapy in babies under 8 kg treated in paediatric intensive care units
	Secondary objective: To compare the use of a novel haemodialysis device with conventional renal replacement therapy using the secondary outcome measures
Outcomes	Primary: Accuracy of fluid removal by technique and compared with prescription
	Secondary: Haemodynamic status Biochemical clearances No of ventilator free days Survival Completion of intended renal replacement therapy course Need for additional vascular or dialysis access Unplanned change in circuits Exposure to blood transfusion Bleeding events Anticoagulant use Parent/guardian experience Staff acceptability and usability of device
Study sites	Birmingham Childrens Hospital Bristol Childrens Hospital Evelina London Childrens Hospital Great Ormond Street Hospital Newcastle (Great North Children’s Hospital and Freeman) University Hospitals Southampton
Participants	Sample: Children 0.8–7.99 kg in PICU who require RRT for renal insufficiency or fluid overload
	Size: approx. 95
Study duration	Approx. 30 months (approx. 20 months recruitment)

PICU, paediatric intensive care unit; RRT, renal replacement therapy.

### Study design

**Figure 1:**
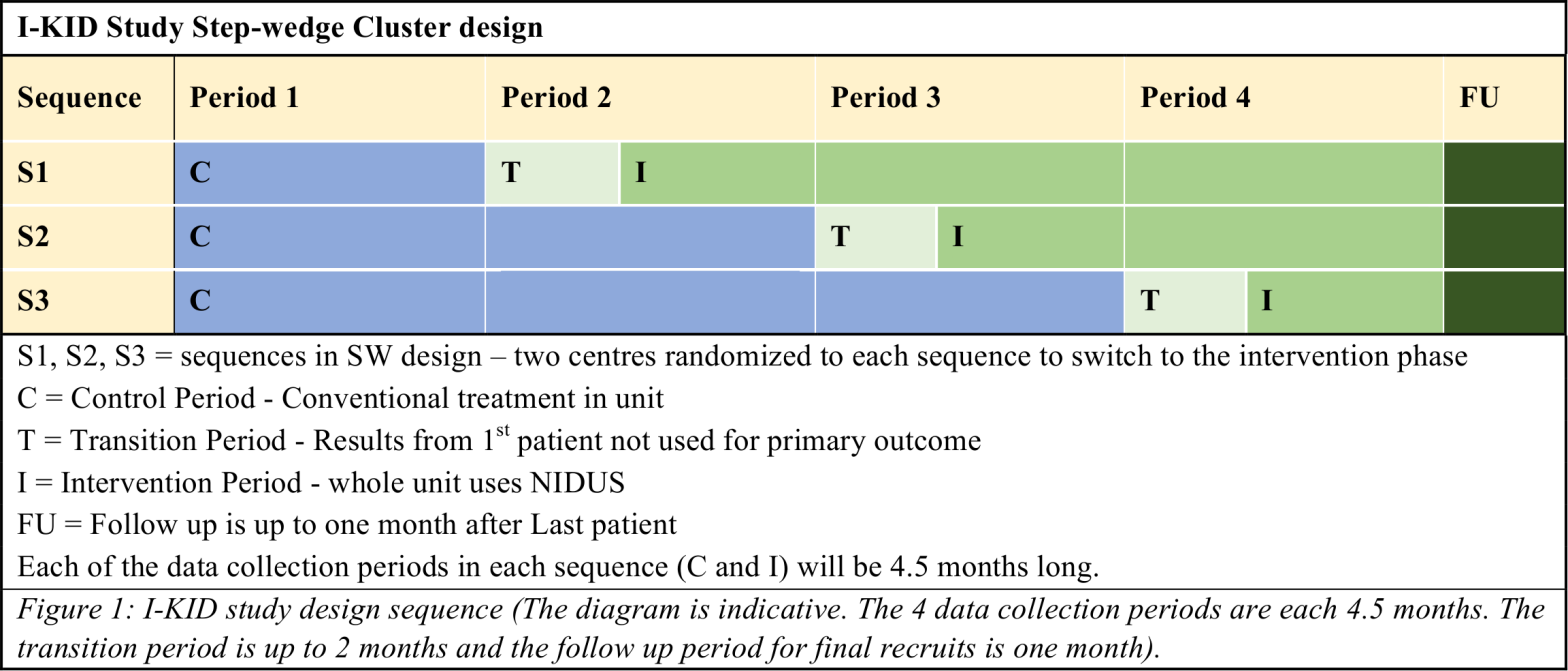
I-KID study design. NIDUS, Newcastle Infant Dialysis Ultrafiltration System; SW, stepped-wedge.

The study uses a cluster randomised standard stepped-wedge (SW) design^25^ with four periods and three sequences (figure 1). The control periods use conventional therapy (PD or CVVH), with NIDUS used in intervention periods. Each site will be trained in setting up and using the NIDUS before switching to an intervention period. The design means that all participating centres will have the chance to use both treatments during the course of the study. PICU nurses will need to be competency assessed before each site can begin using the intervention; 24 hours on call nurse/clinician is provided from Newcastle for telephone support. Using an SW design permits the phased training on NIDUS and allows within-centre comparisons to contribute to the treatment estimate.

### Randomisation

Past records suggested that Great Ormond Street Hospital, Evelina and Southampton (the large centres) treat substantially more patients for RRT than Birmingham, Bristol and Newcastle (the small centres). To avoid large imbalances between the sequences, random permutation in R software was used to allocate one large and one small centre to each sequence. The statistician was blind to the identities of the centres during the allocation.

### Sample selection and outcomes

**Figure 2:**
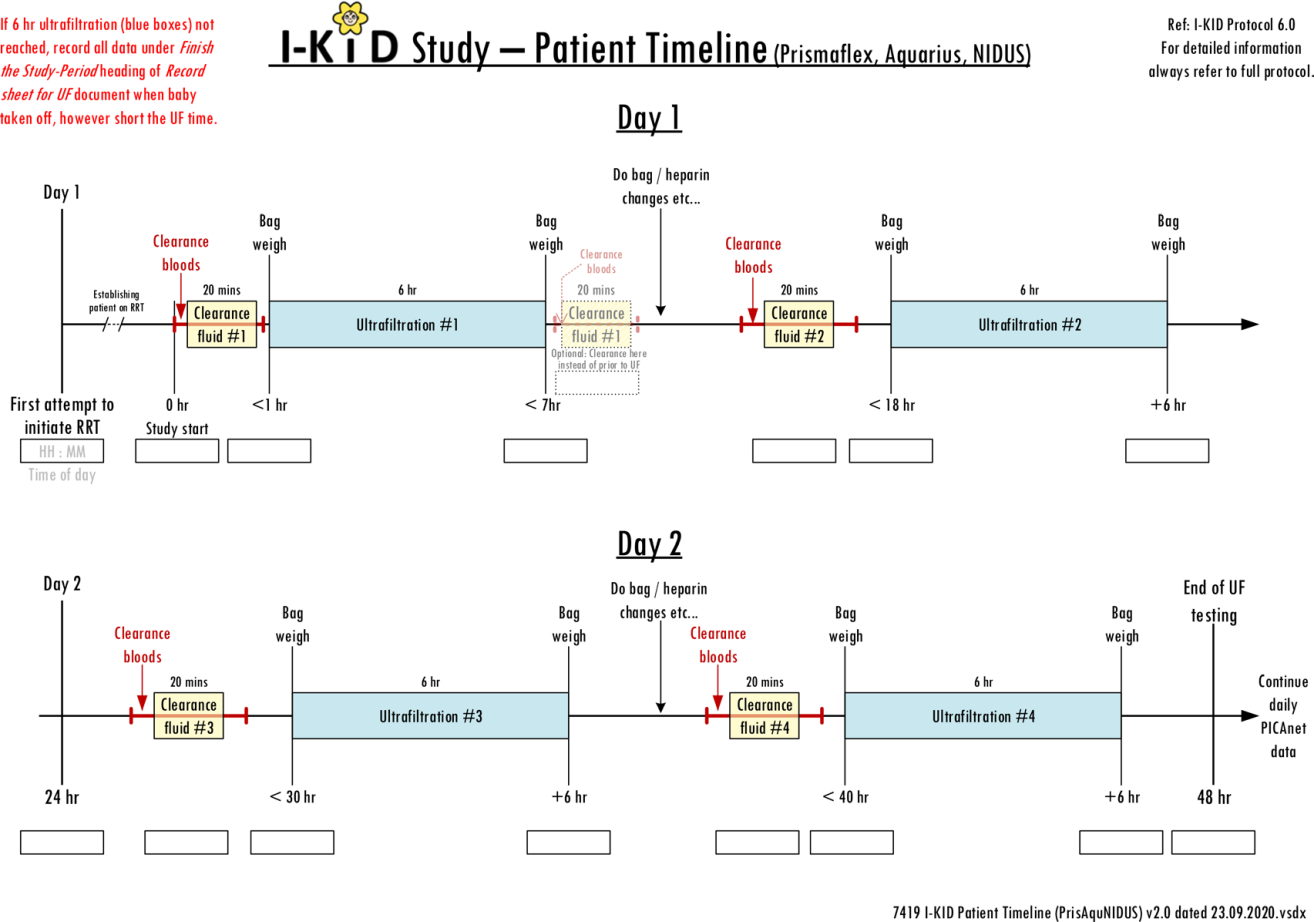
Patient data collection timeline: haemodialysis/filtration devices.

**Figure 3:**
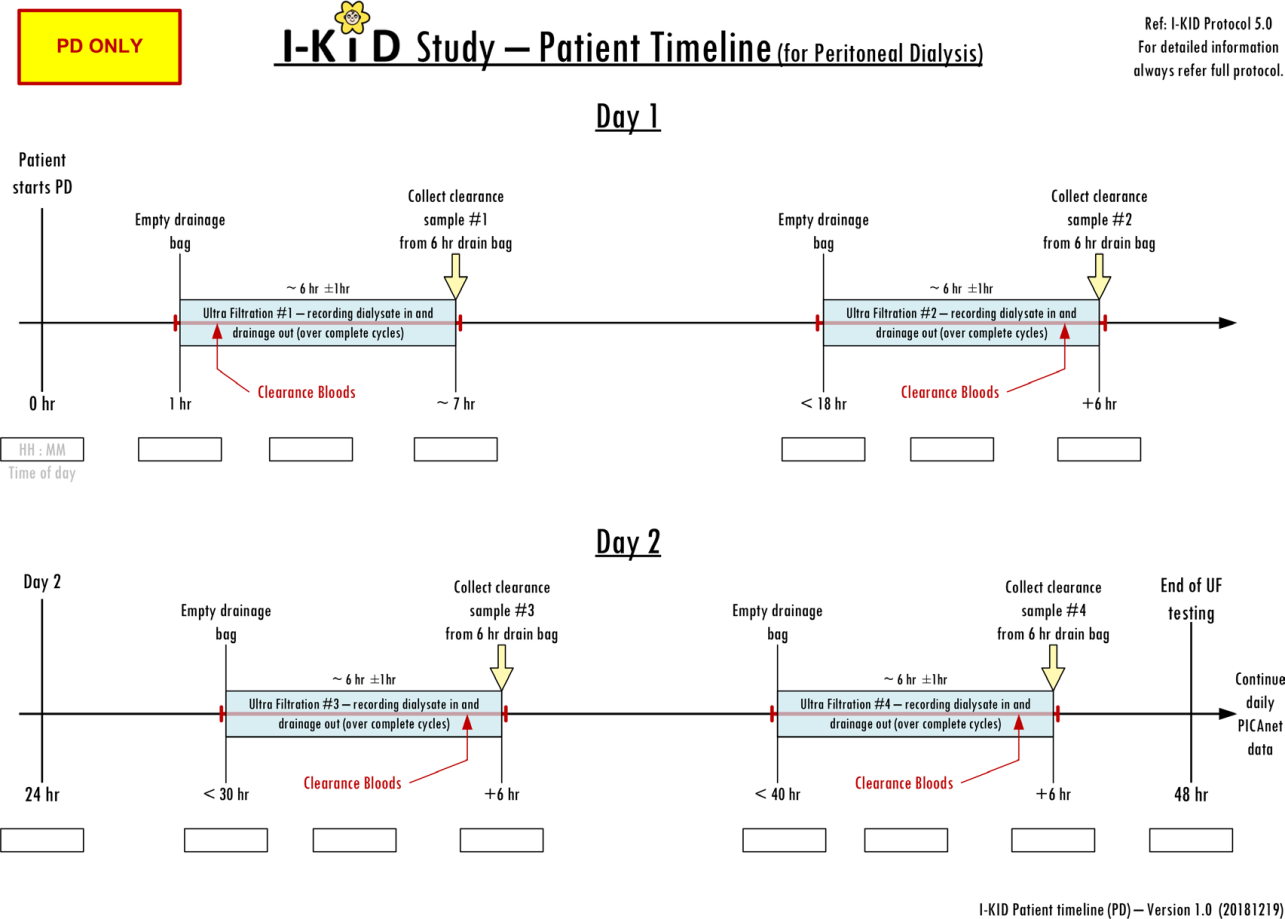
Patient data collection timeline: peritoneal dialysis. PD, peritoneal dialysis.

The study (summarised in table 1, box 1) will be conducted in six PICUs. Inclusion and exclusion criteria are shown in box 2. RRT use and events such as access line changes and blood transfusions will be recorded via the established daily PICANet enhanced renal audit reporting system.^11^ The weight of the dialysate bags will be measured pre and post dialysis to enable accuracy of fluid removal to be calculated and clearances calculated from measurement of blood and dialysate fluid urea, phosphate and enzymatic creatinine (figures 2 and 3). No additional samples will be taken from the patient for the purposes of this study—only results from routine tests and waste dialysate are needed.

Box 1 **I-KID study timeline**

1–3 months: site setup and study procedure training.

4–24 months: case recruitment.

Training for use of Newcastle Infant Dialysis Ultrafiltration System takes place in the weeks leading up to crossover from control to intervention by in-person sessions and ‘dummy’ set up and running of devices. Written instructions, pictorial users guides and short film clips were created and accessed by scannable QRS code to ensure up to date versions were used. In-person sessions were supplemented and replaced with videolink sessions to comply with COVID-19 pandemic-related restriction and refresher training offered as requested by sites and post COVID-19 pandemic shut down of all research activity March–November 2020.

25–30 months: site close down visits, statistical analysis of data, writing reports and to begin dissemination of results to the scientific, medical and nursing community as well as to parent/public interest groups.

1–30 months: management: monthly formal trial management group meetings (minuted) take place in person and by videolink.

Site communication: monthly informal site discussions (documented summary) for principal investigators and site research and clinical teams to share experience and questions take place throughout via phone and videolink.

Box 2 **I-KID inclusion and exclusion criteria**

**Inclusion criteria**

- Patients in paediatric intensive care unit (PICU) with a body weight of 0.8–7.99 kg kg (note: includes estimated body weight in emergency situation) who require continuous renal replacement therapy (RRT) for acute renal insufficiency or fluid overload as part of their standard clinical care.
- Person with legal parental responsibility for the patient provides written informed consent for the patient to take part in the study.*

*This may be after the patient has started dialysis in an emergency situation so as not to delay treatment.

**Exclusion criteria**

- Patient with known chronic renal failure already on established adequate RRT (this exclusion should not apply when chronic RRT has failed and patient requires acute RRT during the PICU admission).
- Patient already established on adequate RRT for whom entry into the study would require additional central venous access, if that access is not required in the view of the clinical team.
- Patient has an underlying (or clinically suspected) diagnosis of a metabolic disease, including hyper ammonaemia and no other indication for RRT.
- Clinician makes a clinical decision that the patient should not receive RRT using Newcastle Infant Dialysis Ultrafiltration System.

Using a study-specific questionnaire, parents/guardians will be asked about their experience and staff will be asked about acceptability and usability of the RRT device. Follow-up/outcome data will be sought from a routine clinic visit approximately 1 month after start of their RRT; this is to establish whether renal recovery took place: this will include clinical information obtained at discharge from PICU.

### Statistical considerations

#### Primary outcome

The primary aim is to compare the precision of the standard therapy and NIDUS to deliver the fluid removal rate prescribed by the treating physician. The primary outcome is based on the first available determination of fluid removal over a period exceeding 1 hour and within the first 48 hours of commencement of RRT: if the observed removal is *X* and the prescribed removal is *A*, the primary outcome is log|*X-A*|. The expected difference of this quantity between the treatment groups is the log of the ratio of the SDs of the determinations by the two methods. The method supposes that *X* follows a normal distribution with mean *A* and hence the variance of the outcome is $\pi^{2}/8$ .

#### Sample size

Historical data suggested that annual recruitment to the large centres would be 14 patients, with 9 patients in each of Bristol and Birmingham and 3 in Newcastle. The sample size was determined to detect a ratio of the SDs under the standard therapy and NIDUS of three, with power at least 80% and two-sided type I error of 5%. A threefold improvement in the precision of fluid removal in this population would be sufficiently marked that it would be likely to change practice. The calculation used the methods in Matthews and Forbes,^26^ adapted to unequal cluster sizes, and found that four periods in the SW design, each of 4.5 months, gave a power of 80% with an assumed intraclass correlation coefficient (ICC) of 0.1% and 84% for an ICC of 0.05. It was believed that these represented conservative choices for the ICC.

#### Secondary outcomes

Fluid removal data aggregated over the duration of RRT, or the first 48 hours if shorter, will be calculated. Biochemical clearances and ventilator-free days while on RRT will be collected. Binary outcomes are: survival (to 30 days and to discharge), haemodynamic status, whether RRT was completed as intended, need for additional vascular access and unplanned change in dialysis circuit, exposure to blood transfusion, bleeding from insertion line and anticoagulant use.

Responses to questionnaires (1) parent/guardian about their experience and (2) to staff regarding acceptability and usability.

### Planned analysis

Analysis of all available data will be on the basis of intention-to-treat. A subgroup analysis will compare NIDUS with conventional CVVH that is, excluding PD. For the latter group the amount of fluid removed (*X*) will be compared with the amount the machine reports to have been removed (*A*).

The primary outcome will be analysed using a linear model with fixed effects for treatment, period and cluster. The use of a fixed rather than random effect for cluster is a response to the interruptions to data collection due to the effect of COVID-19 on PICUs and the subsequent difficulty in defining a suitable dispersion structure. Sensitivity analyses will use a generalised estimating equation and will assess the assumptions about *X*. If these are untenable then *X-A* will be modelled directly, with treatment dependent variances for the error terms. The above linear model will be applied to the non-binary secondary outcomes. Binary outcomes will be analysed using generalised mixed models if possible but using simple tabulations if more sophisticated analyse are infeasible. Questionnaire data will be tabulated by treatment.

### Ethics and dissemination

This study is taking place in a high-risk group of sick infants.

### Parent and public involvement

The NIDUS device was invented because of parental demand in the face of limited options for dialysing their children. Feedback was sought from parents with children on dialysis in Newcastle and the Newcastle University Consumer Research Panel on the study design and parent information. The design uses cluster randomisation for reasons of safety, ethics and acceptability: randomisation by centre, rather than by patient, has been supported by a research consumer group and parents who thought that individual consent for the dialysis method would add to families’ stress and anxiety, and they would tend to defer to their clinician for advice. Individual consent is sought for collection and recording of information.

### Consent

Study information sheets, produced in collaboration with parents, are provided to parents/guardians of all eligible patients. Tailored consent is obtained appropriate to the phase of the study.

Parent and coapplicant CB has been involved in the study development from the start to ensure that methods are acceptable and sensitive. He took part in multiple teleconference discussions and spoke at the study launch event, and along with other interested parents will take part in dissemination of findings.

A level of urgency to recruit, consent and initiate RRT without compromising the patients’ health raises ethical concerns^27^ and delayed consent is accepted following CONNECT best practice^28^; consent from bereaved parents may be sought using the bereaved parent/guardian information sheet and consent form. Discussion with individual parents and the parents on the trial steering committee demonstrated how important they felt this study would be and they approved the use of delayed consent and inclusion of bereaved families.

### Recruitment

Recruitment, which commenced in December 2018, has been more rapid in the control period (62 total) than the intervention (ongoing) and the study has been impacted by the effects of the COVID-19 pandemic on NHS workload and staff availability. There have been three pauses to recruitment (May/June 2019; September/October 2019; November/December 2020) instigated urgently by the chief investigator related to problems identified at sites with the device and tubing/filter sets, to allow investigation and resolution. Medicines and Healthcare products Regulatory Agency (MHRA) temporarily withdrew notice of no objection in March 2020 while awaiting further information from the study team about anticoagulation, which coincided with the COVID-19 pandemic and recruitment to all non-COVID studies stopped; it was reinstated with additional clarification and no changes. Post-COVID-19 restart was in October 2020.

### Protocol changes

The study was initially submitted as a clinical trial, however, due to delays in obtaining CE marking, the study was submitted as a clinical investigation of a medical device in 2017 requiring a change in protocol. Other protocol changes related to the use of deferred consent in emergency situations, and production of a modified parent information sheet and consent form for bereaved families (details in online supplemental file).

10.1136/bmjpo-2021-001224.supp1 Supplementary data

### Results dissemination

Results will be disseminated through publications, conference proceedings and via parent and research newsletters. On overview of results will be provided to families who have taken part.

### Safety reporting

All adverse events (AEs), other than those considered consistent with the usual clinical pattern for patients requiring RRT in PICU and observed device deficiency are collected and recorded. All serious AEs for this study, whether considered device/procedure related or not will be reported to the MHRA in line with regulatory requirements.

### Study oversight

The study is managed by a TMG at Newcastle Clinical Trials Unit (NCTU), with oversight from study sponsor, trial steering and data monitoring committees. A safety subcommittee reviews all safety reports. Data will be handled, computerised and stored in accordance with the Data Protection Act 2018. NCTU will be responsible for the study database and data management procedures.

## Supplementary Material

Author's manuscript

## Data Availability

Data sharing not applicable as no datasets generated and/or analysed for this study. Protocol paper-ongoing data collection.
