# Supplementary material for: I-KID study protocol: evaluation of efficacy, outcomes and safety of a new infant haemodialysis and ultrafiltration machine in clinical use: a randomised clinical investigation using a cluster stepped-wedge design
Source: BMJ Paediatr Open. 2021 Oct 18;5(1):e001224. doi: 10.1136/bmjpo-2021-001224 (PMC8524285; doi:10.1136/bmjpo-2021-001224)
Supplement: Supplementary data [file bmjpo-2021-001224supp001.pdf]

Protocol	Amendments
V1.0 07/12/2015	**Original REC Submission**
V2.0 14/12/2017<br>Notice of no objection 16/02/2018	**Submitted to the MHRA as a Clinical Investigation**
V3.0 22/12/2017<br><br>Approved 21/02/2018	**Submitted to REC and HRA as a Clinical Investigation and included the following changes**<br>**1.** Nidus confirmed as a non-CE marked device<br>**2.** Submission changed to a clinical investigation not CTIMP<br>**3.** Changes to protocol including changes to consent process for two parents with conflicting views on consent<br>**4.** Changes to patient information sheet, for both the control and intervention arms and changes to formatting of consent form.<br>**5.** Addition of site: Evelina London Children's Hospital
V4.0 and V5.0<br>28/09/2018<br><br>Approved 31/10/2018	**Submitted to REC, HRA and MHRA and included the following changes. The MHRA requested updates to the changes therefore protocol version 4.0 was not approved. The changes resulted in the approved protocol V5.0**<br>Changes to Protocol and Patient information sheets to include the following:<br>**1.** Deferred consent in emergency situations<br>**2.** Inclusion of an estimated body weight in emergency situations<br>**3.** Process of using Nidus on compassionate grounds<br>**4.** Safety reporting<br>**5.** Update to patient information sheets to reflect the new data protection requirements
V6.0 15/02/2020<br><br>Approved 27/08/2020	**Submitted to REC, HRA and MHRA and included the following changes.**<br>**1.** Updates to protocol for clarification of the wording<br>**2.** PIS and Consent form documents for Bereaved Parents<br>**3.** Updates to documents from Nidus to NIDUS to match the trademark.
