# Supplementary material for: I-KID study protocol: evaluation of efficacy, outcomes and safety of a new infant haemodialysis and ultrafiltration machine in clinical use: a randomised clinical investigation using a cluster stepped-wedge design
Source: BMJ Paediatr Open. 2021 Oct 18;5(1):e001224. doi: 10.1136/bmjpo-2021-001224 (PMC8524285; doi:10.1136/bmjpo-2021-001224)
Supplement: Author's manuscript [file bmjpo-2021-001224.draft_revisions.pdf]

BMJ Paediatrics Open

BMJ Paediatrics Open is committed to open peer review. As part of this commitment we make the peer review history of every article we publish publicly available.

When an article is published we post the peer reviewers' comments and the authors' responses online. We also post the versions of the paper that were used during peer review. These are the versions that the peer review comments apply to.

The versions of the paper that follow are the versions that were submitted during the peer review process. They are not the versions of record or the final published versions. They should not be cited or distributed as the published version of this manuscript.

BMJ Paediatrics Open is an open access journal and the full, final, typeset and author-corrected version of record of the manuscript is available on our site with no access controls, subscription charges or pay-per-view fees (http://bmjpaedsopen.bmj.com).

If you have any questions on BMJ Paediatrics Open's open peer review process please email info.bmjpo@bmj.com

BMJ Paediatrics Open

The I-KID Study Protocol: Evaluation of Efficacy, Outcomes and Safety of a New Infant Haemodialysis and Ultrafiltration Machine in Clinical Use: a Randomised Clinical Investigation using a Cluster Stepped-wedge Design.

Journal:	*BMJ Paediatrics Open*
Manuscript ID	bmjpo-2021-001224.R1
Article Type:	Protocol
Date Submitted by the Author:	28-Jul-2021

Complete List of Authors:	Lambert, Heather; Great North Children's Hospital, Paediatric Nephrology; Newcastle University,<br>Sharma, Shriya; Newcastle University, Faculty of Medical Sciences<br>Matthews, John; Newcastle University, Department of Mathematics, Statistics & Physics and Population Health Sciences Institute<br>Group, I-KID Protocol Development Group

Keywords:	Nephrology, Neonatology, Technology

SCHOLARONE™
Manuscripts

I, the Submitting Author has the right to grant and does grant on behalf of all authors of the Work (as defined in the below author licence), an exclusive licence and/or a non-exclusive licence for contributions from authors who are: i) UK Crown employees; ii) where BMJ has agreed a CC-BY licence shall apply, and/or iii) in accordance with the terms applicable for US Federal Government officers or employees acting as part of their official duties; on a worldwide, perpetual, irrevocable, royalty-free basis to BMJ Publishing Group Ltd ("BMJ") its licensees and where the relevant Journal is co-owned by BMJ to the co-owners of the Journal, to publish the Work in this journal and any other BMJ products and to exploit all rights, as set out in our licence.

The Submitting Author accepts and understands that any supply made under these terms is made by BMJ to the Submitting Author unless you are acting as an employee on behalf of your employer or a postgraduate student of an affiliated institution which is paying any applicable article publishing charge ("APC") for Open Access articles. Where the Submitting Author wishes to make the Work available on an Open Access basis (and intends to pay the relevant APC), the terms of reuse of such Open Access shall be governed by a Creative Commons licence – details of these licences and which Creative Commons licence will apply to this Work are set out in our licence referred to above.

Other than as permitted in any relevant BMJ Author's Self Archiving Policies, I confirm this Work has not been accepted for publication elsewhere, is not being considered for publication elsewhere and does not duplicate material already published. I confirm all authors consent to publication of this Work and authorise the granting of this licence.

Confidential: For Review Only

Title: The I-KID Study Protocol: Evaluation of Efficacy, Outcomes and Safety of a New Infant Haemodialysis and Ultrafiltration Machine in Clinical Use: a Randomised Clinical Investigation using a Cluster Stepped-wedge Design.

Authors

Heather J Lambert[1], Shriya Sharma[2] & John NS Matthews[3] on behalf of the IKID Protocol Development Group (see below for membership)

1 The Great North Children's Hospital, Newcastle Hospitals NHS Trust

2 Newcastle Clinical Trials Unit, Newcastle University

3 Department of Mathematics, Statistics & Physics and Population Health Sciences Institute, Newcastle University

Key words Infant Kidney Dialysis Filtration

What is known about this subject

Babies in paediatric intensive care unit (PICU) may develop acute renal failure and require therapeutic support.

Renal replacement options for babies are limited because of their small size and limitations of technology.

Current haemodialysis and filtration systems in use in UK are not recommended or licensed for children under 8kg.

What this study hopes to add

Renal replacement methods for infants under 8kg in PICU will be compared.

The efficacy, outcomes and safety of a new infant haemodialysis device will be assessed.

Usability of the new device in normal clinical settings outside of the development centre will be examined.

Word count 2688

Confidential: For Review Only

Abstract

Introduction: The I-KID study aims to determine the clinical efficacy, outcomes and safety of a novel non-CE-marked infant haemodialysis machine, the Newcastle Infant Dialysis Ultrafiltration System (NIDUS), compared to currently available therapy in the UK. NIDUS is specifically designed for renal replacement therapy in small babies between 0.8 and 8 kilograms.

Methods and analysis: The clinical investigation is taking place in six UK centres. This is a randomised clinical investigation using a cluster stepped-wedge design. The study aims to recruit 95 babies requiring renal replacement therapy in paediatric intensive care units over 20 months.

Ethics and Dissemination: The study has high parent and public involvement at all stages in its design and parents will be involved in dissmination of results to parents and professionals via publications, conference proceedings and newletters. The study has has ethics permissions from Tyne and Wear South Research Ethics Committee.

Registration

IRAS ID number: 170481
MHRA Reference: CI/2017/0066
ISRCT Number: 13787486
CPMS ID number: 36558
NHS REC reference: 16/NE/0008
Eudamed number: CIV-GB-18-02-023105

Link to full protocol v6.0: https://fundingawards.nihr.ac.uk/award/14/23/26

Confidential: For Review Only

Introduction

Young babies requiring renal replacement therapy (RRT) present specific therapeutic challenges because of their small size and the current technology available. Publications indicate similar problems faced by clinicians worldwide who use adult devices because of lack of alternatives, and the need for solutions including improved device technology [1,2].

This clinical investigation protocol is designed to determine the clinical efficacy, outcomes and safety of a novel non-CE-marked infant haemodialysis machine, the Newcastle Infant Dialysis Ultrafiltration System (NIDUS), compared to currently available RRT in the UK. NIDUS is designed for use in babies between 0.8 and 8 kilograms (kg).There is evidence from a previous single centre pilot study to anticipate NIDUS has the potential to contribute significant benefits to small babies needing RRT [3].

The proposed clinical investigation is a result of a multicentre collaboration between clinicians, scientists, academics, with significant parent and public involvement, throughout its development; working with a manufacturing company, Allmed. The results will have potential to change clinical practice.

The NIDUS machine uses a smaller circuit volume than current devices. Pilot data from the development centre has suggested management of fluid overload and renal failure is possible for small infants, with the potential for reduced exposure to blood products, and more precise control of ultrafiltration and dialysis [3]. Nurses have reported ease of use of the NIDUS within the design centre but this requires evaluation in standard clinical environments.

Background

There are several populations of babies requiring RRT. Those included in this study are unwell infants in paediatric intensive care units (PICU), who mostly do not have intrinsic renal disease and therefore have good potential for renal recovery. Many are post-operative, especially post cardiac surgery, whose major problem is an acute kidney insult, fluid overload and poor urine output, and others who are septic or have renal failure as part of multi-organ failure. Although mortality and morbidity in PICU varies and is related to the underlying diagnosis, survival of babies in PICU is worse in those with fluid overload [4] or needing RRT [5], of whom up to 20-40% may die [5,6,7,8,9]. RRT is supportive until kidney recovery and although most survivors are independent of RRT at discharge from PICU, data on chronic renal sequelae are lacking. Children requiring RRT in PICU have been reported to have longer length of stay and required more days of ventilator support [8]. There are over 200 infants per year in the UK receiving treatment with continuous RRT in PICU [10,11].

Some babies will be excluded – for example, those with an inborn error of metabolism such as urea cycle defects causing hyperammonaemia, as they require emergency, very rapid removal of toxic metabolites by higher than normal dialysis clearances [12], and babies with severe intrinsic renal disease, which is often congenital, who are usually treated with chronic peritoneal dialysis (PD) at home.

Confidential: For Review Only

Current Renal Replacement Therapies

PD is used frequently to support infants after open-heart surgery [5,13]. PD is technically simpler than haemodialysis (HD); there is no lower size limit but complications are common in the smallest patients [4]. Ultrafiltration (UF) is unpredictable, and chemical clearance less efficient, especially in unstable babies who develop splanchnic vasoconstriction and who also risk developing necrotising enterocolitis. This renders PD impossible, as does abdominal surgery and congenital abdominal wall defects. Larger critically ill infants with multi-organ failure are often treated with a variety of continuously delivered haemodialysis (HD) modalities (continuous renal replacement therapy, CRRT) [4,5,9]. Vascular access for HD modalities including Continuous Veno-Venous Haemofiltration (CVVH) is problematic as the size of central venous line (CVL) required for adequate blood flow is disproportionately large for the size of the baby especially when a double lumen line is needed.

Whilst there are no randomised controlled trials in infants, publications indicate recurring themes of difficulties with vascular access and blood flows, fluid balance, rapid clotting, loss of circuits and hypotensive episodes at initiation [6,7,8,9].

Conventional HD and CRRT machines in the UK are used in PICU unlicensed as they are CE marked for use in adults and bigger children. Manufacturers quote fluid balance control as ±30 ml/hour [14], and they therefore are not licensed for babies weighing <8 kg (or approved for use in children of <20 kg in the US). The recommended minimum 7-French, dual-lumen vascular access lines and continuous 40 ml/minute blood flows are difficult to achieve in the smallest babies. Their relatively large circuit volume (50-70ml) produces sudden dilution of blood on commencing treatment if crystalloid primed, and increases the risk of anaemia with circuit loss. Hypotension on connection is a reported problem [6,15,16]. Blood priming risks exposing the baby abruptly to aberrant chemical and pH changes, which are reduced by pre-dialysing the circuit [17]. Exposure to blood transfusions increases the risk of developing tissue-type sensitisation which may be important if renal function does not recover and renal or other solid organ transplant is considered in the future.

There is one CE marked new device for smaller children, the CARPEDIEM, which is not yet available in the UK to enable comparisons [18,19], others, notably in USA, have adapted other adult devices like Aquadex [9,20].

NIDUS Technology

In 1995, a group in Newcastle designed a novel HD circuit, which operated by different principles i.e. by syringes, and uncoupled the baby's blood flow capacity from the requirements of the dialysis filter [21]. In 2005, they reported the results of automating this as a miniaturised machine (circuit volume less than 10 ml), with which four babies weighing under 4 kg were treated, using a single-lumen access line, and without the need for blood-priming [22]. This device was subsequently developed into NIDUS [3] which is used as the intervention device in the I-KID study. This clinical investigation will contribute to the current knowledge base and further understanding of the effects of RRT and address the need for improved technology to provide RRT effectively and safely for small babies [1,23,24].

Safety monitoring is an important focus of this study. The NIDUS makes a downloadable constant recording of all activity data including volumes, flows, pressures, alarms and

Confidential: For Review Only

response to alarms so any alarm or event, however small, can be subsequently analysed. The NIDUS potentially provides a safer way of performing RRT on babies by using a novel circuit that allows precise ultrafiltrate control thus reducing the potential for errors in ultrafiltrate removal that would be trivial for larger children but are not for a baby. Its small circuit volume (<10 ml) potentially avoids the need for blood priming with stored blood which has associated immediate risks and long-term risks of developing sensitising antibodies.

Confidential: For Review Only

Methods and analysis

The study aims to evaluate the efficacy and precision of NIDUS in ultrafiltration fluid removal and monitor adverse effects of RRT including use of blood product transfusion (Table1). It will also generate a safety profile in the application of NIDUS in the clinical environment.

Study Design

The study uses a cluster-randomised standard stepped-wedge (SW) design [25] with four periods and three sequences (Figure 1). The control periods use conventional therapy (PD or CVVH), with NIDUS used in intervention periods. Each site will be trained in setting up and using the NIDUS before switching to an intervention period. The design means that all participating centres will have the chance to use both treatments during the course of the study. PICU nurses will need to be competency assessed before each site can begin using the intervention; 24h on call nurse/clinician is provided from Newcastle for telephone support. Using a SW design permits the phased training on NIDUS and allows within-centre comparisons to contribute to the treatment estimate.

Randomisation

Past records suggested that GOSH, Evelina and Southampton (the large centres) treat substantially more patients for RRT than Birmingham, Bristol & Newcastle (the small centres). To avoid large imbalances between the sequences, random permutation in R software was used to allocate one large and one small centre to each sequence. The statistician was blind to the identities of the centres during the allocation.

Sample selection and outcomes

The study (summarised in Tables 1 and 2) will be conducted in six PICUs. Inclusion and exclusion criteria are shown in Table 3. RRT use and events such as access line changes and blood transfusions will be recorded via the established daily PICANet enhanced renal audit reporting system [11]. The weight of the dialysate bags will be measured pre and post dialysis to enable accuracy of fluid removal to be calculated and clearances calculated from measurement of blood and dialysate fluid urea, phosphate and enzymatic creatinine (Figures

2,3). No additional samples will be taken from the patient for the purposes of this study – only results from routine tests and waste dialysate are needed.

Using a study-specific questionnaire, parents/guardians will be asked about their experience and staff will be asked about acceptability and usability of the RRT device. Follow up/outcome data will be sought from a routine clinic visit approximately 1 month after start of their RRT; this is to establish whether renal recovery took place: this will include clinical information obtained at discharge from PICU.

Statistical considerations

Primary outcome

The primary aim is to compare the precision of the standard therapy and NIDUS to deliver the fluid removal rate prescribed by the treating physician. The primary outcome is based on the first available determination of fluid removal over a period exceeding one hour and within the first 48 hours of commencement of RRT: if the observed removal is X and the prescribed removal is A, the primary outcome is $\log|X\text{-}A|$. The expected difference of this quantity between the treatment groups is the log of the ratio of the standard deviations (SDs) of the determinations by the two methods. The method supposes that X follows a normal distribution with mean A and hence the variance of the outcome is $\pi^2/8$.

Sample size

Historical data suggested that annual recruitment to the large centres would be 14 patients, with 9 patients in each of Bristol and Birmingham and 3 in Newcastle. The sample size was determined to detect a ratio of the SDs under the standard therapy and NIDUS of three, with power at least 80% and two-sided type I error of 5%. A three-fold improvement in the precision of fluid removal in this population would be sufficiently marked that it would be likely to change practice. The calculation used the methods in Matthews & Forbes [26], adapted to unequal cluster sizes, and found that four periods in the SW design, each of 4.5 months, gave a power of 80% with an assumed Intraclass Correlation, ICC,of 0.1 and 84% for an ICC of 0.05. It was believed that these represented conservative choices for the ICC.

Secondary Outcomes

Fluid removal data aggregated over the duration of RRT, or the first 48 hours if shorter, will be calculated. Biochemical clearances and ventilator-free days while on RRT will be collected. Binary outcomes are: survival [to 30 days and to discharge], haemodynamic status, whether RRT was completed as intended, need for additional vascular access and unplanned change in dialysis circuit, exposure to blood transfusion, bleeding from insertion line and anticoagulant use.

Responses to questionnaires i) parent/guardian about their experience and ii) to staff regarding acceptability and usability.

Planned Analysis

Confidential: For Review Only

Analysis of all available data will be on the basis of intention-to-treat. A subgroup analysis will compare NIDUS with conventional CVVH i.e. excluding PD. For the latter group the amount of fluid removed (*X*) will be compared with the amount the machine reports to have been removed *(A)*.

The primary outcome will be analysed using a linear model with fixed effects for treatment, period and cluster. The use of a fixed rather than random effect for cluster is a response to the interruptions to data collection due to the effect of COVID-19 on PICUs and the subsequent difficulty in defining a suitable dispersion structure. Sensitivity analyses will use a generalized estimating equation and will assess the assumptions about *X*. If these are untenable then *X-A* will be modelled directly, with treatment dependent variances for the error terms. The above linear model will be applied to the non-binary secondary outcomes. Binary outcomes will be analysed using generalized mixed models if possible but using simple tabulations if more sophisticated analyse are infeasible. Questionnaire data will be tabulated by treatment.

Ethics and Dissemination

This study is taking place in a high-risk group of sick infants.

Parent and public involvement

The NIDUS device was invented because of parental demand in the face of limited options for dialysing their children. Feedback was sought from parents with children on dialysis in Newcastle and the Newcastle University Consumer Research Panel on the study design and parent information. The design uses cluster-randomisation for reasons of safety, ethics, and acceptability: randomisation by centre, rather than by patient, has been supported by a Research Consumer Group and parents who thought that individual consent for the dialysis method would add to families' stress and anxiety, and they would tend to defer to their clinician for advice. Individual consent is sought for collection and recording of information.

Consent

Study Information sheets, produced in collaboration with parents, are provided to parents/guardians of all eligible patients. Tailored consent is obtained appropriate to the phase of the study.

Parent and co-applicant CB has been involved in the study development from the start to ensure that methods are acceptable and sensitive. He took part in multiple teleconference discussions and spoke at the study launch event, and along with other interested parents will take part in dissemination of findings.

A level of urgency to recruit, consent and initiate RRT without compromising the patients' health raises ethical concerns [27] and delayed consent is accepted following CONNECT best practice [28]; consent from bereaved parents may be sought using the bereaved parent/guardian information sheet and consent form. Discussion with individual parents and the parents on the Trial Steering Committee demonstrated how important they felt this study would be and they approved the use of delayed consent and inclusion of bereaved families.

https://mc.manuscriptcentral.com/bmjpo
Confidential: For Review Only

Recruitment

Recruitment, which commenced in December 2018 has been more rapid in the control period (62 total) than the intervention (ongoing) and the study has been impacted by the effects of the COVID pandemic on NHS workload and staff availability. There have been 3 pauses to recruitment (May/June 2019; September/October 2019; November/December 2020) instigated urgently by the Chief Investigator related to problems identified at sites with the device and tubing/filter sets, to allow investigation and resolution. MHRA temporarily withdrew notice of no objection in March 2020 whilst awaiting further information from the study team about anticoagulation, which coincided with the COVID pandemic and recruitment to all non-COVID studies stopped; it was reinstated with additional clarification and no changes. Post COVID restart was in October 2020.

Protocol Changes

The study was initially submitted as a clinical trial, however due to delays in obtaining CE marking, the study was submitted as a Clinical Investigation of a Medical Device in 2017 requiring a change in protocol. Other protocol changes related to the use of deferred consent in emergency situations, and production of a modified parent information sheet and consent form for bereaved families.

Results dissemination

Results will be disseminated through publications, conference proceedings and via parent and research newsletters. On overview of results will be provided to families who have taken part.

Ethics Permission

Favourable ethical opinion was obtained from Tyne and Wear South Research Ethics Committee. A letter of no objection was obtained from the Medicines and Healthcare products Regulatory Agency (MHRA).

Safety Reporting

All adverse events (AE), other than those considered consistent with the usual clinical pattern for patients requiring RRT in PICU, and observed Device Deficiency (DD) are collected and recorded. All serious AEs for this study, whether considered device/procedure related or not will be reported to the MHRA in line with regulatory requirements.

Study Oversight

The study is managed by a TMG at Newcastle Clinical Trials Unit (NCTU), with oversight from Study Sponsor, Trial Steering and Data Monitoring Committees. A Safety subcommittee reviews all safety reports. Data will be handled, computerised and stored in

Confidential: For Review Only

accordance with the Data Protection Act 2018. NCTU will be responsible for the study database and data management procedures.

Funding statement

Study funded by: Efficacy and Mechanism Evaluation Programme (EME) Funder Reference: 14/23/26

Sponsored by: The Newcastle Upon Tyne Hospitals NHS Foundation Trust Sponsor reference: 7419

Acknowledgements

We thank the parents and families of babies recruited to the study for their important contribution, particularly at a such stressful and anxious time when their baby is unwell.

We thank the members of the public and parents involved in reviewing the I-KID study and contributing to the study design and parent information.

We acknowledge the work, time and enthusiasm of the site research and clinical staff in making this study happen, their continuous support and for enabling restart to recruitment following interruption due to the COVID pandemic.

We acknowledge the work of Michael Drinnan, Jim Wightman and Alison Bray from the Northern Medical Physics and Clinical Engineering Directorate, NUTH in the technical support and development of the NIDUS device.

Confidential: For Review Only

Tables and Figures

Table 1: I-KID study summary

Table 2: I-KID Study Timeline

Table 3: I-KID Inclusion and Exclusion Criteria

Figure 1: I-KID Study design

Figure 2: Patient Data Collection Timeline: Haemodialysis/filtration devices

Figure 3: Patient Data Collection Timeline: Peritoneal Dialysis

Table 1: I-KID study summary

**Design**	A multi-centre, randomised clinical investigation using a cluster stepped-wedge design
**Study interventions**	Control: current renal replacement therapy (either Peritoneal Dialysis or Continuous Veno-Venous Haemofiltration)
	Experimental intervention: renal replacement therapy using the Newcastle Infant Dialysis Ultrafiltration System (NIDUS)
**Objectives**	Primary: To compare the use of a novel haemodialysis device with conventional renal replacement therapy in babies under 8kg treated in Paediatric Intensive Care Units
	Secondary objective: To compare the use of a novel haemodialysis device with conventional renal replacement therapy using the secondary outcome measures
**Outcomes**	Primary: Accuracy of fluid removal by technique and compared with prescription
	Secondary:<br>• Haemodynamic status<br>• Biochemical clearances<br>• Number of ventilator free days<br>• Survival<br>• Completion of intended renal replacement therapy course<br>• Need for additional vascular or dialysis access<br>• Unplanned change in circuits<br>• Exposure to blood transfusion<br>• Bleeding events<br>• Anticoagulant use<br>• Parent/Guardian experience

Confidential: For Review Only

	• Staff acceptability and usability of device
**Study sites**	• Birmingham Childrens Hospital<br>• Bristol Childrens Hospital<br>• Evelina London Childrens Hospital<br>• Great Ormond Street Hospital<br>• Newcastle (Great North Children's Hospital and Freeman)<br>• University Hospitals Southampton
**Participants**	Sample: Children 0.8kg to 7.99 kg in PICU who require RRT for renal insufficiency or fluid overload
	Size: approx 95
**Study duration**	Approx. 30 months (approx. 20 months recruitment)

Confidential: For Review Only

Table 2: I-KID Study Timeline

1-3 months: site set up and study procedure training

4-24 months: case recruitment

Training for use of NIDUS takes place in the weeks leading up to crossover from contol to intervention by inperson sessions and "dummy" set up and running of devices. Written instructions, pictorial users guides and short film clips were created and accessed by scannable QRS code to ensure up to date versions were used. In-person sessions were supplemented and replaced with videolink sessions to comply with COVID pandemic related restriction and refresher training offered as requested by sites and post COVID pandemic shut down of all research activity March-November 2020.

25-30 months: site close down visits, statistical analysis of data, writing reports and to begin dissemination of results to the scientific, medical and nursing community as well as to parent/public interest groups.

1-30m

Management: monthly formal Trial Management Group meetings (minuted) take place in person and by videolink.

Site communication: monthly informal site discussions (documented summary) for Principal Investigators and site research and clinical teams to share experience and questions take place throughout via phone and videolink.

Confidential: For Review Only

Table 3: I-KID Inclusion and Exclusion Criteria

Inclusion criteria

- Patients in PICU with a body weight of 0.8 kg – 7.99 kg (note: includes estimated body weight in emergency situation) who require continuous RRT for acute renal insufficiency or fluid overload as part of their standard clinical care.
- *Person with legal parental responsibility (PR) for the patient provides written informed consent for the patient to take part in the study.

*This may be after the patient has started dialysis in an emergency situation so as not to delay treatment.

Exclusion criteria

- Patient with known chronic renal failure already on established adequate RRT (This exclusion should not apply when chronic RRT has failed and patient requires acute RRT during the PICU admission).
- Patient already established on adequate RRT for whom entry into the study would require additional central venous access, if that access is not required in the view of the clinical team.
- Patient has an underlying (or clinically suspected) diagnosis of a metabolic disease, including hyper ammonaemia and no other indication for RRT.
- Clinician makes a clinical decision that the patient should not receive RRT using NIDUS.

Confidential: For Review Only

Confidential: For Review Only

Author's contributions

- HJL is the chief investigator and conceived the study, led its design and co-ordination and drafted the manuscript.
- MGC conceived the original NIDUS device concept and jointly designed the study and contributed to the writing of the manuscript.
- JNSM jointly designed the study and led the planned statistical analysis and contributed to the writing of the manuscript.
- CB participated in the design of the study and the preparation of parent information and aids in planning and disseminating information about the study.
- DH, QM, JS, KM, CW, RP, LW, JC, RA, EM, DG, JP, AN, HD participated in the design and development of the study and contributed to review of the manuscript.
- MG, SH, NW contributed statistical expertise to protocol development.
- MW, JB, IG & RH contributed to device engineering and study development.
- The NCTU team JWa, SS, E-MH, AS, KR and KN assisted in preparation of the clinical trial protocol to comply with the type recognised by governance and legislative bodies and assisted with the preparation of information sheets and consent forms and overseeing protocol amendments. RW led design of data management.
- DC, JS, RS, VE, JO, CPa contributed to protocol development of training, support and user instruction.
- CPr, KT contributed to protocol development.
- All authors read and approved the final manuscript.

I-KID Protocol Development Group

Heather Lambert (HL)

John Matthews (JNSM)

Malcolm Coulthard (MGC)

Daljit Hothi (DH)

Quen Mok (QM)

Jon Smith (JS)

Kevin Morris (KM)

Lucy Wirz (LW)

Jean Crosier (JC)

Rachel Agbeko (RA)

Chris Boucher (CB)

Elaine McColl (EM)

David Grant (DG)

John Pappachan (JP)

https://mc.manuscriptcentral.com/bmjpo
For Review Only

Andrew Nyman (AN)

Heather Duncan (HD)

Claire Westrope (CW)

PICAnet: Roger Parslow (RP), Richard Feltbower (RW)

Northern Medical Physics & Clinical Engineering Directorate: Michael Whitaker (MW), Joseph Bulmer (JB), Ina Guri (IG), Rebecca Harrison (RH).

Newcastle University Biostatistics Research Group: John Matthews (JM), Michael Grayling (MG), Shaun Hiu (SH), Nina Wilson (NW)

Newcastle Clinical Trials Unit: Jenn Walker (JWa), Shriya Sharma (SS), Ruth Wood (RW), Eva-Maria Holstein (E-MH), Alison Steel (AS), Katherine Rennie (KR) and Karen Nicholson (KN)

NUTH specialist renal nurses: Denise Chisholm (DS), Jayne Straker (JS), Rachel Steel (RS), Vikki Emmet (VE), Julie Office (JO), Christine Pattinson (CPa)

NUTH Sponsor and Project management: Chris Price (CPr), Kelsie Thomas (KT)

Competing interests

MGC is named on the patent and will receive some royalties if the device goes into commercial production.

The Newcastle upon Tyne NHS Foundation Trust (NUTH) will receive some royalties if the device goes into commercial production.

Allmed are providing 17 NIDUS devices for loan to sites for the I-KID study and for potential compassionate use in the post-study period. NIDUS consumables are purchased as per normal clinical care.

There are no other competing interests declared.

Confidential: For Review Only

References

1. Tal L, Angelo JR, Akcan-Arikan A. Neonatal extracorporeal renal replacement therapy-a routine renal support modality. Pediatr Nephrol March 2016 (31):2013-2015

2. Lee ST, Cho H. Fluid overload and outcomes in neonates receiving continuous renal replacement therapy. Pediatr Nephrol March 2016 (31):2145-2152

3. Coulthard MG, Crosier J, Griffiths C, Smith J, Drinnan M, Whitaker M, Beckwith R, Matthews JN, Flecknell P, Lambert HJ. Haemodialysing babies weighing <8 kg with the Newcastle infant dialysis and ultrafiltration system (Nidus): comparison with peritoneal and conventional haemodialysis. Pediatr Nephrol. 2014 Oct;29(10):1873-81.

4. Sutherland SM, Alexander SR. Continuous renal replacement therapy in children. Pediatr Nephrol. 2012 Nov;27(11):2007-2016.

5. Westrope CA, Fleming S, Kapetanstrataki M, Parslow RC, Morris KP. Renal Replacement Therapy in the Critically Ill Child. Pediatr Crit Care Med. 2018 Mar;19(3):210-217.

6. Santiago MJ, López-Herce J, Urbano J, Solana MJ, del Castillo J, Ballestero Y, Botrán M, Bellón JM. Clinical course and mortality risk factors in critically ill children requiring continuous renal replacement therapy. Intensive Care Med. 2010 May;36(5):843-9.

7. Askenazi DJ, Goldstein SL, Koralkar R, Fortenberry J, Baum M, Hackbarth R, Blowey D, Bunchman TE, Brophy PD, Symons J, Chua A, Flores F, Somers MJ. Continuous renal replacement therapy for children ≤10 kg: a report from the prospective pediatric continuous renal replacement therapy registry. J Pediatr. 2013 Mar;162(3):587-592.

8. Jetton JG, Boohaker LJ, Sethi SK, Wazir S, Rohatgi S, Soranno DE, Chishti AS, Woroniecki R, Mammen C, Swanson JR, Sridhar S, Wong CS, Kupferman JC, Griffin RL, Askenazi DJ; Neonatal Kidney Collaborative (NKC). Incidence and outcomes of neonatal acute kidney injury (AWAKEN): a multicentre, multinational, observational cohort study. Lancet Child Adolesc Health. 2017 Nov;1(3):184-194.

9. Menon S, Broderick J, Munshi R, Dill L, DePaoli B, Fathallah-Shaykh S, Claes D, Goldstein SL, Askenazi DJ. Kidney Support in Children using an Ultrafiltration Device: A Multicenter, Retrospective Study. Clin J Am Soc Nephrol. 2019 Oct 7;14(10):1432-1440.

10. Paediatric Intensive Care Audit Network Annual Report 2010 - 2012 (published September 2013): Universities of Leeds and Leicester. [Online] Available from http://www.picanet.org.uk/Audit/Annual-Reporting/PICANet Annual Report 2013 Summary.pdf

11. Paediatric Intensive Care Audit Network Annual Report 2020 (published February 2021): Universities of Leeds and Leicester: https://www.picanet.org.uk/wp-content/uploads/sites/25/2021/02/PICANet2020_AnnualReportSummary_v1.0.pdf.

12. Westrope C, Morris K, Burford D, Morrison G. Continuous hemofiltration in the control of neonatal hyperammonemia: a 10-year experience. Pediatr Nephrol. 2010 Sep;25(9):1725-30.

For peer review only

13. Bojan M, Gioanni S, Vouhé PR, Journois D, Pouard P. Early initiation of peritoneal dialysis in neonates and infants with acute kidney injury following cardiac surgery is associated with a significant decrease in mortality. Kidney Int. 2012 Aug;82(4):474-81.

14. Diaa M. Prismaflex user's manual. http://www.scribdcom/doc/141000459/Prismaflex

15. Hothi DK, St George-Hyslop C, Geary D, Bohn D, Harvey E. Continuous renal replacement therapy (CRRT) in children using the AQUARIUS. Nephrol Dial Transplant. 2006 Aug;21(8):2296-300.

16. Sohn YB, Paik KH, Cho HY, Kim SJ, Park SW, Kim ES, Chang YS, Park WS, Choi YH, Jin DK. Continuous renal replacement therapy in neonates weighing less than 3 kg. Korean J Pediatr. 2012 Aug;55(8):286-92.

17. Hackbarth RM, Eding D, Gianoli Smith C, Koch A, Sanfilippo DJ, Bunchman TE. Zero balance ultrafiltration (Z-BUF) in blood-primed CRRT circuits achieves electrolyte and acid-base homeostasis prior to patient connection. Pediatr Nephrol. 2005 Sep;20(9):1328-33.

18. Ronco C, Garzotto F, Brendolan A, Zanella M, Bellettato M, Vedovato S, Chiarenza F, Ricci Z, Goldstein SL. Continuous renal replacement therapy in neonates and small infants: development and first-in-human use of a miniaturised machine (CARPEDIEM). Lancet. 2014 May 24;383(9931):1807-13.

19. Lorenzin A, Garzotto F, Alghisi A, Neri M, Galeano D, Aresu S, Pani A, Vidal E, Ricci Z, Murer L, Goldstein SL, Ronco C. CVVHD treatment with CARPEDIEM: small solute clearance at different blood and dialysate flows with three different surface area filter configurations. Pediatr Nephrol. 2016 Oct;31(10):1659-65.

20. Askenazi D, Ingram D, White S, Cramer M, Borasino S, Coghill C, Dill L, Tenney F, Feig D, Fathallah-Shaykh S. Smaller circuits for smaller patients: improving renal support therapy with Aquadex™. Pediatr Nephrol. 2016 May;31(5):853-60.

21. Coulthard MG, Sharp J. Haemodialysis and ultrafiltration in babies weighing under 1000 g. Arch Dis Child Fetal Neonatal Ed. 1995 Nov;73(3):F162-5.

22. Everdell NL, Coulthard MG, Crosier J, Keir MJ. A machine for haemodialysing very small infants. Pediatr Nephrol. 2005 May;20(5):636-43.

23. Hothi DK. Designing technology to meet the therapeutic demands of acute renal injury in neonates and small infants. Pediatr Nephrol. 2014 Oct;29(10):1869-71.

24. Laskin BL, Foster BJ. Extracorporeal therapy for the smallest children. Lancet. 2014 May 24;383(9931):1785-6.

25. Hussey MA, Hughes JP. Design and analysis of stepped wedge cluster randomized trials. Contemp Clin Trials. 2007 Feb;28(2):182-91.

26. Matthews JNS, Forbes AB. Stepped wedge designs: insights from a design of experiments perspective. Stat Med. 2017 Oct 30;36(24):3772-3790.

27. Brierley J, Larcher V. Compassionate and innovative treatments in children: a proposal for an ethical framework. Arch Dis Child. 2009 Sep;94(9):651-4.

28. University of Liverpool. Research without prior consent (deferred consent) in trials investigating the emergency treatment of critically ill children: CONNECT study guidance.

For peer review only

2015.
https://www.liverpool.ac.uk/media/livacuk/iphs/Research,without,prior,consent,in,trials,investigating,the,emergency,treatment,of,critically,ill,children,CONNECT,guidance,July,2015.pdf

Confidential: For Review Only

Figure 1: I-KID Study design

I-KID Study Step-wedge Cluster design

**Sequence**	**Period 1**	**Period 2**	**Period 3**	**Period 4**	**FU**
**S1**	**C**	**T I**			
**S2**	**C**		**T I**		
**S3**	**C**			**T I**	

S1, S2, S3 = sequences in SW design – two centres randomized to each sequence to switch to the intervention phase

C = Control Period - Conventional treatment in unit

T = Transition Period - Results from 1st patient not used for primary outcome

I = Intervention Period - whole unit uses NIDUS

FU = Follow up is up to one month after Last patient

Each of the data collection periods in each sequence (C and I) will be 4.5 months long.

Figure 1: I-KID study design sequence (The diagram is indicative. The 4 data collection periods are each 4.5 months. The transition period is up to 2 months and the follow up period for final recruits is one month).

Confidential: For Review Only

If 6 hr ultrafiltration (blue boxes) not reached, record all data under *Finish the Study-Period* heading of *Record sheet for UF* document when baby taken off, however short the UF time.

I-KID Study – Patient Timeline (Prismaflex, Aquarius, NIDUS)

Ref: I-KID Protocol 6.0
For detailed information always refer to full protocol.

Day 1

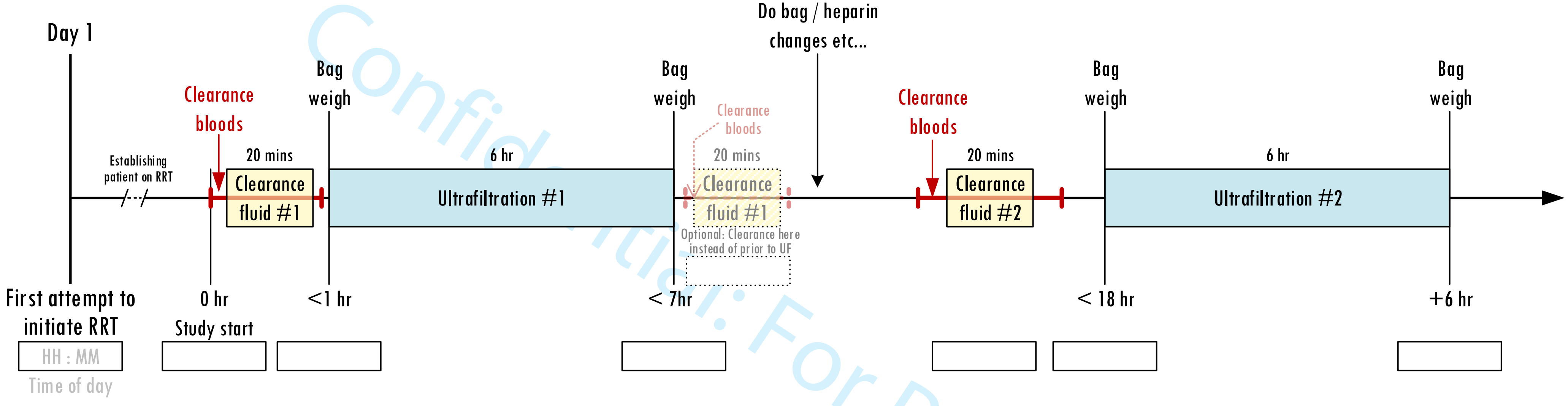

Day 2

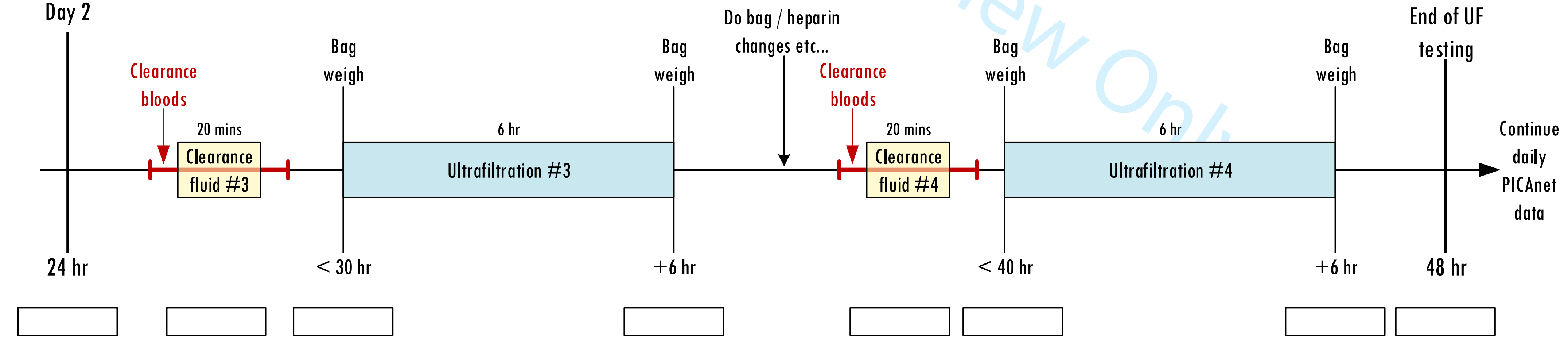

Confidential: For Review Only

7419 I-KID Patient Timeline (PrisAquNIDUS) v2.0 dated 23.09.2020.vsdx

BMJ Paediatrics Open

PD ONLY

I-KiD Study – Patient Timeline (for Peritoneal Dialysis)

Ref: I-KID Protocol 5.0
For detailed information always refer full protocol.

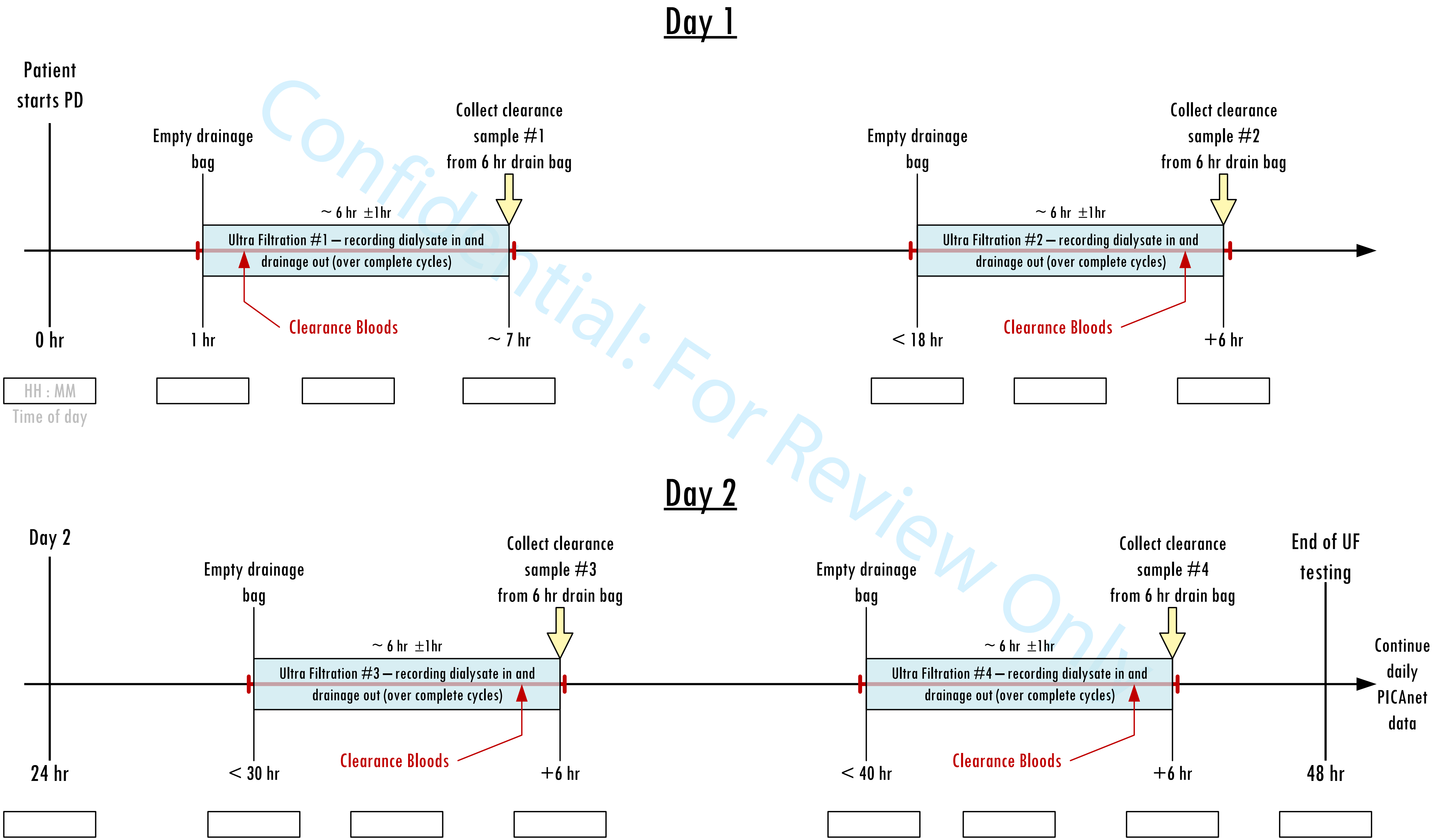

Confidential: For Review Only

I-KID Patient timeline (PD) – Version 1.0 (20181219)

Protocol	Amendments
V1.0 07/12/2015	**Original REC Submission**
V2.0 14/12/2017<br>Notice of no objection 16/02/2018	**Submitted to the MHRA as a Clinical Investigation**
V3.0 22/12/2017<br><br>Approved 21/02/2018	**Submitted to REC and HRA as a Clinical Investigation and included the following changes**<br>**1.** Nidus confirmed as a non-CE marked device<br>**2.** Submission changed to a clinical investigation not CTIMP<br>**3.** Changes to protocol including changes to consent process for two parents with conflicting views on consent<br>**4.** Changes to patient information sheet, for both the control and intervention arms and changes to formatting of consent form.<br>**5.** Addition of site: Evelina London Children's Hospital
V4.0 and V5.0 28/09/2018<br><br>Approved 31/10/2018	**Submitted to REC, HRA and MHRA and included the following changes. The MHRA requested updates to the changes therefore protocol version 4.0 was not approved. The changes resulted in the approved protocol V5.0**<br>Changes to Protocol and Patient information sheets to include the following:<br>**1.** Deferred consent in emergency situations<br>**2.** Inclusion of an estimated body weight in emergency situations<br>**3.** Process of using Nidus on compassionate grounds<br>**4.** Safety reporting<br>**5.** Update to patient information sheets to reflect the new data protection requirements
V6.0 15/02/2020<br><br>Approved 27/08/2020	**Submitted to REC, HRA and MHRA and included the following changes.**<br>**1.** Updates to protocol for clarification of the wording<br>**2.** PIS and Consent form documents for Bereaved Parents<br>**3.** Updates to documents from Nidus to NIDUS to match the trademark.

For peer review only

https://mc.manuscriptcentral.com/bmjpo

BMJ Paediatrics Open

The I-KID Study Protocol: Evaluation of Efficacy, Outcomes and Safety of a New Infant Haemodialysis and Ultrafiltration Machine in Clinical Use: a Randomised Clinical Investigation using a Cluster Stepped-wedge Design.

Journal:	*BMJ Paediatrics Open*
Manuscript ID	bmjpo-2021-001224
Article Type:	Protocol
Date Submitted by the Author:	20-Jul-2021

Complete List of Authors:	Lambert, Heather; Great North Children's Hospital, Paediatric Nephrology; Newcastle University,<br>Sharma, Shriya; Newcastle University, Faculty of Medical Sciences<br>Matthews, John; Newcastle University, Department of Mathematics, Statistics & Physics and Population Health Sciences Institute<br>Group, I-KID Protocol Development Group

Keywords:	Nephrology, Neonatology, Technology

SCHOLARONE™
Manuscripts

I, the Submitting Author has the right to grant and does grant on behalf of all authors of the Work (as defined in the below author licence), an exclusive licence and/or a non-exclusive licence for contributions from authors who are: i) UK Crown employees; ii) where BMJ has agreed a CC-BY licence shall apply, and/or iii) in accordance with the terms applicable for US Federal Government officers or employees acting as part of their official duties; on a worldwide, perpetual, irrevocable, royalty-free basis to BMJ Publishing Group Ltd ("BMJ") its licensees and where the relevant Journal is co-owned by BMJ to the co-owners of the Journal, to publish the Work in this journal and any other BMJ products and to exploit all rights, as set out in our licence.

The Submitting Author accepts and understands that any supply made under these terms is made by BMJ to the Submitting Author unless you are acting as an employee on behalf of your employer or a postgraduate student of an affiliated institution which is paying any applicable article publishing charge ("APC") for Open Access articles. Where the Submitting Author wishes to make the Work available on an Open Access basis (and intends to pay the relevant APC), the terms of reuse of such Open Access shall be governed by a Creative Commons licence – details of these licences and which Creative Commons licence will apply to this Work are set out in our licence referred to above.

Other than as permitted in any relevant BMJ Author's Self Archiving Policies, I confirm this Work has not been accepted for publication elsewhere, is not being considered for publication elsewhere and does not duplicate material already published. I confirm all authors consent to publication of this Work and authorise the granting of this licence.

Confidential: For Review Only

Title: The I-KID Study Protocol: Evaluation of Efficacy, Outcomes and Safety of a New Infant Haemodialysis and Ultrafiltration Machine in Clinical Use: a Randomised Clinical Investigation using a Cluster Stepped-wedge Design.

Authors

Heather J Lambert[1], Shriya Sharma[2] & John NS Matthews[3] on behalf of the IKID Protocol Development Group (see below for membership)

1 The Great North Children's Hospital, Newcastle Hospitals NHS Trust

2 Newcastle Clinical Trials Unit, Newcastle University

3 Department of Mathematics, Statistics & Physics and Population Health Sciences Institute, Newcastle University

Key words Infant Kidney Dialysis Filtration

What is known about this subject

Babies in paediatric intensive care unit (PICU) may develop acute renal failure and require therapeutic support.

Renal replacement options for babies are limited because of their small size and limitations of technology.

Current haemodialysis and filtration systems in use in UK are not recommended or licensed for children under 8kg.

What this study hopes to add

Renal replacement methods for infants under 8kg in PICU will be compared.

The efficacy, outcomes and safety of a new infant haemodialysis device will be assessed.

Usability of the new device in normal clinical settings outside of the development centre will be examined.

Word count 2500

Confidential: For Review Only

Abstract

Introduction: The I-KID study aims to determine the clinical efficacy, outcomes and safety of a novel non-CE-marked infant haemodialysis machine, the Newcastle Infant Dialysis Ultrafiltration System (NIDUS), compared to currently available therapy in the UK. NIDUS is specifically designed for renal replacement therapy in small babies between 0.8 and 8 kilograms.

Methods and analysis: The clinical investigation is taking place in six UK centres. This is a randomised clinical investigation using a cluster stepped-wedge design. The study aims to recruit 95 babies requiring renal replacement therapy in paediatric intensive care units over 20 months.

Ethics and Dissemination: The study has high parent and public involvement at all stages in its design and parents will be involved in dissmination of results to parents and professionals via publications, conference proceedings and newletters. The study has has ethics permissions from Tyne and Wear South Research Ethics Committee.

Registration

IRAS ID number: 170481
MHRA Reference: CI/2017/0066
ISRCT Number: 13787486
CPMS ID number: 36558
NHS REC reference: 16/NE/0008
Eudamed number: CIV-GB-18-02-023105

Link to full protocol v6.0: https://fundingawards.nihr.ac.uk/award/14/23/26

Confidential: For Review Only

Introduction

Young babies requiring renal replacement therapy (RRT) present specific therapeutic challenges because of their small size and the current technology available. Publications indicate similar problems faced by clinicians worldwide who use adult devices because of lack of alternatives, and the need for solutions including improved device technology.

This clinical investigation protocol is designed to determine the clinical efficacy, outcomes and safety of a novel non-CE-marked infant haemodialysis machine, the Newcastle Infant Dialysis Ultrafiltration System (NIDUS), compared to currently available RRT in the UK. NIDUS is designed for use in babies between 0.8 and 8 kilograms (kg).There is evidence from a previous single centre pilot study to anticipate NIDUS has the potential to contribute significant benefits to small babies needing RRT [1] .

The proposed clinical investigation is a result of a multicentre collaboration between clinicians, scientists, academics, with significant parent and public involvement, throughout its development; working with a manufacturing company, Allmed. The results will have potential to change clinical practice.

The NIDUS machine uses a smaller circuit volume than current devices. Pilot data from the development centre has suggested management of fluid overload and renal failure is possible for small infants, with the potential for reduced exposure to blood products, and more precise control of ultrafiltration and dialysis [1]. Nurses have reported ease of use of the NIDUS within the design centre but this requires evaluation in standard clinical environments.

Background

There are several populations of babies requiring RRT. Those included in this study are unwell infants in paediatric intensive care units (PICU), who mostly do not have intrinsic renal disease and therefore have good potential for renal recovery. Many are post-operative, especially post cardiac surgery, whose major problem is an acute kidney insult, fluid overload and poor urine output, and others who are septic or have renal failure as part of multi-organ failure. Although mortality and morbidity in PICU varies and is related to the underlying diagnosis, survival of babies in PICU is worse in those with fluid overload [2] or needing RRT [3], of whom up to 20-40% may die [3,4,5,6,7]. RRT is supportive until kidney recovery and although most survivors are independent of RRT at discharge from PICU, data on chronic renal sequelae are lacking. Children requiring RRT in PICU have been reported to have longer length of stay and required more days of ventilator support [6]. There are over 200 infants per year in the UK receiving treatment with continuous RRT in PICU [8a 8b].

Some babies will be excluded – for example, those with an inborn error of metabolism such as urea cycle defects causing hyperammonaemia, as they require emergency, very rapid removal of toxic metabolites by higher than normal dialysis clearances [9], and babies with severe intrinsic renal disease, which is often congenital, who are usually treated with chronic peritoneal dialysis (PD) at home.

Confidential: For Review Only

Confidential: For Review Only

Current Renal Replacement Therapies

PD is used frequently to support infants after open-heart surgery [3,10]. PD is technically simpler than haemodialysis (HD); there is no lower size limit but complications are common in the smallest patients [2]. Ultrafiltration (UF) is unpredictable, and chemical clearance less efficient, especially in unstable babies who develop splanchnic vasoconstriction and who also risk developing necrotising enterocolitis. This renders PD impossible, as does abdominal surgery and congenital abdominal wall defects. Larger critically ill infants with multi-organ failure are often treated with a variety of continuously delivered haemodialysis (HD) modalities (continuous renal replacement therapy, CRRT) [2,3,7]. Vascular access for HD modalities including Continuous Veno-Venous Haemofiltration (CVVH) is problematic as the size of central venous line (CVL) required for adequate blood flow is disproportionately large for the size of the baby especially when a double lumen line is needed.

Whilst there are no randomised controlled trials in infants, publications indicate recurring themes of difficulties with vascular access and blood flows, fluid balance, rapid clotting, loss of circuits and hypotensive episodes at initiation [4,5,6,7].

Conventional HD and CRRT machines in the UK are used in PICU unlicensed as they are CE marked for use in adults and bigger children. Manufacturers quote fluid balance control as ±30 ml/hour [11], and they therefore are not licensed for babies weighing <8 kg (or approved for use in children of <20 kg in the US). The recommended minimum 7-French, dual-lumen vascular access lines and continuous 40 ml/minute blood flows are difficult to achieve in the smallest babies. Their relatively large circuit volume (50-70ml) produces sudden dilution of blood on commencing treatment if crystalloid primed, and increases the risk of anaemia with circuit loss. Hypotension on connection is a reported problem [4,12,13]. Blood priming risks exposing the baby abruptly to aberrant chemical and pH changes, which are reduced by pre-dialysing the circuit [14]. Exposure to blood transfusions increases the risk of developing tissue-type sensitisation which may be important if renal function does not recover and renal or other solid organ transplant is considered in the future.

There is one CE marked new device for smaller children, the CARPEDIEM, which is not yet available in the UK to enable comparisons [15,16], others, notably in USA, have adapted other adult devices like Aquadex [7,17].

NIDUS Technology

In 1995, a group in Newcastle designed a novel HD circuit, which operated by different principles i.e. by syringes, and uncoupled the baby's blood flow capacity from the requirements of the dialysis filter [18]. In 2005, they reported the results of automating this as a miniaturised machine (circuit volume less than 10 ml), with which four babies weighing under 4 kg were treated, using a single-lumen access line, and without the need for blood-priming [19]. This device was subsequently developed into NIDUS [1] which is used as the intervention device in the I-KID study. This clinical investigation will contribute to the current knowledge base and further understanding of the effects of RRT and address the need for improved technology to provide RRT effectively and safely for small babies [20,21].

Safety monitoring is an important focus of this study. The NIDUS makes a downloadable constant recording of all activity data including volumes, flows, pressures, alarms and

response to alarms so any alarm or event, however small, can be subsequently analysed. The NIDUS potentially provides a safer way of performing RRT on babies by using a novel circuit that allows precise ultrafiltrate control thus reducing the potential for errors in ultrafiltrate removal that would be trivial for larger children but are not for a baby. Its small circuit volume (<10 ml) potentially avoids the need for blood priming with stored blood which has associated immediate risks and long-term risks of developing sensitising antibodies.

Methods and analysis

The study aims to evaluate the efficacy and precision of NIDUS in ultrafiltration fluid removal and monitor adverse effects of RRT including use of blood product transfusion (Table1). It will also generate a safety profile in the application of NIDUS in the clinical environment.

Study Design

The study uses a cluster-randomised standard stepped-wedge (SW) design [22] with four periods and three sequences (Figure 1). The control periods use conventional therapy (PD or CVVH), with NIDUS used in intervention periods. Each site will be trained in setting up and using the NIDUS before switching to an intervention period. The design means that all participating centres will have the chance to use both treatments during the course of the study. PICU nurses will need to be competency assessed before each site can begin using the intervention; 24h on call nurse/clinician will be provided from Newcastle for telephone support.

Using a SW design permits the phased training on NIDUS and allows within-centre comparisons to contribute to the treatment estimate.

Randomisation

Past records suggested that GOSH, Evelina and Southampton (the large centres) treat substantially more patients for RRT than Birmingham, Bristol & Newcastle (the small centres). To avoid large imbalances between the sequences, random permutation in R software was used to allocate one large and one small centre to each sequence. The statistician was blind to the identities of the centres during the allocation.

Sample selection and outcomes

The study (summarised in Tables 1 and 2) will be conducted in six PICUs. Inclusion and exclusion criteria are shown in Table 3. RRT use and events such as access line changes and blood transfusions will be recorded via the established daily PICANet enhanced renal audit reporting system [8,26]. The weight of the dialysate bags will be measured pre and

Confidential: For Review Only

post dialysis to enable accuracy of fluid removal to be calculated and clearances calculated from measurement of blood and dialysate fluid urea, phosphate and enzymatic creatinine (Figure 3). No additional samples will be taken from the patient for the purposes of this study – only results from routine tests and waste dialysate are needed.

Using a study-specific questionnaire, parents/guardians will be asked about their experience and staff will be asked about acceptability and usability of the RRT device. Follow up/outcome data will be sought from a routine clinic visit approximately 1 month after start of their RRT; this is to establish whether renal recovery took place: this will include clinical information obtained at discharge from PICU.

Confidential: For Review Only

Statistical considerations

Primary outcome

The primary aim is to compare the precision of the standard therapy and NIDUS to deliver the fluid removal rate prescribed by the treating physician. The primary outcome is based on the first available determination of fluid removal over a period exceeding one hour and within the first 48 hours of commencement of RRT: if the observed removal is X and the prescribed removal is A, the primary outcome is $\log|X\text{-}A|$. The expected difference of this quantity between the treatment groups is the log of the ratio of the standard deviations (SDs) of the determinations by the two methods. The method supposes that X follows a normal distribution with mean A and hence the variance of the outcome is $\pi^2/8$.

Sample size

Historical data suggested that annual recruitment to the large centres would be 14 patients, with 9 patients in each of Bristol and Birmingham and 3 in Newcastle. The sample size was determined to detect a ratio of the SDs under the standard therapy and NIDUS of three, with power at least 80% and two-sided type I error of 5%. A three-fold improvement in the precision of fluid removal in this population would be sufficiently marked that it would be likely to change practice. The calculation used the methods in Matthews & Forbes [23], adapted to unequal cluster sizes, and found that four periods in the SW design, each of 4.5 months, gave a power of 80% with an assumed Intraclass Correlation, ICC,of 0.1 and 84% for an ICC of 0.05. It was believed that these represented conservative choices for the ICC.

Secondary Outcomes

Fluid removal data aggregated over the duration of RRT, or the first 48 hours if shorter, will be calculated. Biochemical clearances and ventilator-free days while on RRT will be collected. Binary outcomes are: survival [to 30 days and to discharge], haemodynamic status, whether RRT was completed as intended, need for additional vascular access and unplanned change in dialysis circuit, exposure to blood transfusion, bleeding from insertion line and anticoagulant use.

Responses to questionnaires i) parent/guardian about their experience and ii) to staff regarding acceptability and usability.

Planned Analysis

Analysis of all available data will be on the basis of intention-to-treat. A subgroup analysis will compare NIDUS with conventional CVVH i.e. excluding PD. For the latter group the amount of fluid removed (*X*) will be compared with the amount the machine reports to have been removed *(A)*.

The primary outcome will be analysed using a linear model with fixed effects for treatment, period and cluster. The use of a fixed rather than random effect for cluster is a response to the interruptions to data collection due to the effect of COVID-19 on PICUs and the subsequent difficulty in defining a suitable dispersion structure. Sensitivity analyses will use a generalized estimating equation and will assess the assumptions about *X*. If these are untenable then *X*-*A* will be modelled directly, with treatment dependent variances for the error terms. The above linear model will be applied to the non-binary secondary outcomes. Binary outcomes will be analysed using generalized mixed models if possible but using simple tabulations if more sophisticated analyse are infeasible. Questionnaire data will be tabulated by treatment.

Ethics and Dissemination

This study is taking place in a high-risk group of sick infants.

Parent and public involvement

The NIDUS device was invented because of parental demand in the face of limited options for dialysing their children. Feedback was sought from parents with children on dialysis in Newcastle and the Newcastle University Consumer Research Panel on the study design and parent information. The design uses cluster-randomisation for reasons of safety, ethics, and acceptability: randomisation by centre, rather than by patient, has been supported by a Research Consumer Group and parents who thought that individual consent for the dialysis method would add to families' stress and anxiety, and they would tend to defer to their clinician for advice. Individual consent is sought for collection and recording of information.

Consent

Study Information sheets, produced in collaboration with parents, are provided to parents/guardians of all eligible patients. Tailored consent is obtained appropriate to the phase of the study.

Parent and co-applicant CB has been involved in the study development from the start to ensure that methods are acceptable and sensitive. He took part in multiple teleconference discussions and spoke at the study launch event, and along with other interested parents will take part in dissemination of findings.

A level of urgency to recruit, consent and initiate RRT without compromising the patients' health further raises ethical concerns [24] and delayed consent will be accepted following CONNECT best practice [25]; consent from bereaved parents may be sought using the bereaved parent/guardian information sheet and consent form. Discussion with individual parents and the parents on the Trial Steering Committee demonstrated how important they

Confidential: For Review Only

felt this study would be and they approved the use of delayed consent and inclusion of bereaved families.

Results dissemination

Results will be disseminated through publications, conference proceedings and via parent and research newsletters. On overview of results will be provided to families who have taken part.

Ethics Permission

Favourable ethical opinion was obtained from Tyne and Wear South Research Ethics Committee. A letter of no objection was obtained from the Medicines and Healthcare products Regulatory Agency (MHRA).

Safety Reporting

All adverse events (AE), other than those considered consistent with the usual clinical pattern for patients requiring RRT in PICU, and observed Device Deficiency (DD) are collected and recorded. All serious AEs for this study, whether considered device/procedure related or not will be reported to the MHRA in line with regulatory requirements.

Study Oversight

The study is managed by a Trial Management Group at Newcastle Clinical Trials Unit (NCTU), with oversight from Study Sponsor, Trial Steering and Data Monitoring Committees. A Safety subcommittee reviews all safety reports.

Data will be handled, computerised and stored in accordance with the Data Protection Act 2018. NCTU will be responsible for the study database and data management procedures.

Funding statement

Study funded by: Efficacy and Mechanism Evaluation Programme (EME) Funder Reference: 14/23/26

Sponsored by: The Newcastle Upon Tyne Hospitals NHS Foundation Trust Sponsor reference: 7419

Acknowledgements

We thank the parents and families of babies recruited to the study for their important contribution, particularly at a such stressful and anxious time when their baby is unwell.

We thank the members of the public and parents involved in reviewing the I-KID study and contributing to the study design and parent information.

Confidential: For Review Only

We acknowledge the work, time and enthusiasm of the site research and clinical staff in making this study happen, their continuous support and for enabling restart to recruitment following interruption due to the COVID pandemic.

We acknowledge the work of Michael Drinnan, Jim Weightman and Alison Bray from the Northern Medical Physics and Clinical Engineering Directorate, NUTH in the technical support and development of the NIDUS device.

Confidential: For Review Only

Tables and Figures

Table 1: I-KID study summary

Table 2: I-KID Study Timeline

Table 3: I-KID Inclusion and Exclusion Criteria

Figure 1: I-KID Study design

Figure 2: Patient Data Collection Timeline

2a Hamodialysis/filtration devices

2b Peritoneal Dialysis

Table 1: I-KID study summary

**Design**	A multi-centre, randomised clinical investigation using a cluster stepped-wedge design
**Study interventions**	Control: current renal replacement therapy (either Peritoneal Dialysis or Continuous Veno-Venous Haemofiltration)
	Experimental intervention: renal replacement therapy using the Newcastle Infant Dialysis Ultrafiltration System (NIDUS)
**Objectives**	Primary: To compare the use of a novel haemodialysis device with conventional renal replacement therapy in babies under 8kg treated in Paediatric Intensive Care Units
	Secondary objective: To compare the use of a novel haemodialysis device with conventional renal replacement therapy using the secondary outcome measures
**Outcomes**	Primary: Accuracy of fluid removal by technique and compared with prescription
	Secondary:<br>• Haemodynamic status<br>• Biochemical clearances<br>• Number of ventilator free days<br>• Survival<br>• Completion of intended renal replacement therapy course<br>• Need for additional vascular or dialysis access<br>• Unplanned change in circuits<br>• Exposure to blood transfusion<br>• Bleeding events<br>• Anticoagulant use<br>• Parent/Guardian experience

Confidential: For Review Only

	• Staff acceptability and usability of device
**Study sites**	• Birmingham Childrens Hospital<br>• Bristol Childrens Hospital<br>• Evelina London Childrens Hospital<br>• Great Ormond Street Hospital<br>• Newcastle (Great North Children's Hospital and Freeman)<br>• University Hospitals Southampton
**Participants**	Sample: Children 0.8kg to 7.99 kg in PICU who require RRT for renal insufficiency or fluid overload
	Size: approx 95
**Study duration**	Approx. 30 months (approx. 20 months recruitment)

Confidential: For Review Only

Table 2: I-KID Study Timeline

1-3 months: site set up and study procedure training

4-24 months: case recruitment
Training for use of NIDUS takes place in the weeks leading up to crossover from contol to intervention by inperson sessions and "dummy" set up and running of devices. Written instructions, pictorial users guides and short film clips were created and accessed by scannable QRS code to ensure up to date versions were used. In-person sessions were supplemented and replaced with videolink sessions to comply with COVID pandemic related restriction and refresher training offered as requested by sites and post COVID pandemic shut down of all research activity March-November 2020.

25-30 months: site close down visits, statistical analysis of data, writing reports and to begin dissemination of results to the scientific, medical and nursing community as well as to parent/public interest groups.

1-30m

Management: monthly formal Trial Management Group meetings (minuted) take place in person and by videolink.

Site communication: monthly informal site discussions (documented summary) for Principal Investigators and site research and clinical teams to share experience and questions take place throughout via phone and videolink.

Confidential: For Review Only

Table 3: I-KID Inclusion and Exclusion Criteria

Inclusion criteria

- Patients in PICU with a body weight of 0.8 kg – 7.99 kg (note: includes estimated body weight in emergency situation) who require continuous RRT for acute renal insufficiency or fluid overload as part of their standard clinical care.
- *Person with legal parental responsibility (PR) for the patient provides written informed consent for the patient to take part in the study.

*This may be after the patient has started dialysis in an emergency situation so as not to delay treatment.

Exclusion criteria

- Patient with known chronic renal failure already on established adequate RRT (This exclusion should not apply when chronic RRT has failed and patient requires acute RRT during the PICU admission).
- Patient already established on adequate RRT for whom entry into the study would require additional central venous access, if that access is not required in the view of the clinical team.
- Patient has an underlying (or clinically suspected) diagnosis of a metabolic disease, including hyper ammonaemia and no other indication for RRT.
- Clinician makes a clinical decision that the patient should not receive RRT using NIDUS.

Confidential: For Review Only

Confidential: For Review Only

Author's contributions

- HJL is the chief investigator and conceived the study, led its design and co-ordination and drafted the manuscript.
- MGC conceived the original NIDUS device concept and jointly designed the study and contributed to the writing of the manuscript.
- JNSM jointly designed the study and led the planned statistical analysis and contributed to the writing of the manuscript.
- CB participated in the design of the study and the preparation of parent information and aids in planning and disseminating information about the study.
- DH, QM, JS, KM, CW, RP, LW, JC, RA, EM, DG, JP, AN, HD participated in the design and development of the study and contributed to review of the manuscript.
- MG, SH, NW contributed statistical expertise to protocol development.
- MW, JB, IG & RH contributed to device engineering and study development.
- The NCTU team JWa, SS, E-MH, AS, KR and KN assisted in preparation of the clinical trial protocol to comply with the type recognised by governance and legislative bodies and assisted with the preparation of information sheets and consent forms and overseeing protocol amendments. RW led design of data management.
- DC, JS, RS, VE, JO, CPa contributed to protocol development of training, support and user instruction.
- CPr, KT contributed to protocol development.
- All authors read and approved the final manuscript.

I-KID Protocol Development Group

Heather Lambert (HL)

John Matthews (JNSM)

Malcolm Coulthard (MGC)

Daljit Hothi (DH)

Quen Mok (QM)

Jon Smith (JS)

Kevin Morris (KM)

Lucy Wirz (LW)

Jean Crosier (JC)

Rachel Agbeko (RA)

Chris Boucher (CB)

Elaine McColl (EM)

David Grant (DG)

John Pappachan (JP)

https://mc.manuscriptcentral.com/bmjpo
For Review Only

Andrew Nyman (AN)

Heather Duncan (HD)

Claire Westrope (CW)

PICAnet: Roger Parslow (RP), Richard Feltbower (RW)

Northern Medical Physics & Clinical Engineering Directorate: Michael Whitaker (MW), Joseph Bulmer (JB), Ina Guri (IG), Rebecca Harrison (RH).

Newcastle University Biostatistics Research Group: John Matthews (JM), Michael Grayling (MG), Shaun Hiu (SH), Nina Wilson (NW)

Newcastle Clinical Trials Unit: Jenn Walker (JWa), Shriya Sharma (SS), Ruth Wood (RW), Eva-Maria Holstein (E-MH), Alison Steel (AS), Katherine Rennie (KR) and Karen Nicholson (KN)

NUTH specialist renal nurses: Denise Chisholm (DS), Jayne Straker (JS), Rachel Steel (RS), Vikki Emmet (VE), Julie Office (JO), Christine Pattinson (CPa)

NUTH Sponsor and Project management: Chris Price (CPr), Kelsie Thomas (KT)

Competing interests

MGC is named on the patent and will receive some royalties if the device goes into commercial production.

The Newcastle upon Tyne NHS Foundation Trust (NUTH) will receive some royalties if the device goes into commercial production.

Allmed are providing 17 NIDUS devices for loan to sites for the I-KID study and for potential compassionate use in the post-study period. NIDUS consumables are purchased as per normal clinical care.

There are no other competing interests declared.

Confidential: For Review Only

References

1. Coulthard MG, Crosier J, Griffiths C, Smith J, Drinnan M, Whitaker M, Beckwith R, Matthews JN, Flecknell P, Lambert HJ. Haemodialysing babies weighing <8 kg with the Newcastle infant dialysis and ultrafiltration system (Nidus): comparison with peritoneal and conventional haemodialysis. Pediatr Nephrol. 2014 Oct;29(10):1873-81.

2. Sutherland SM, Alexander SR. Continuous renal replacement therapy in children. Pediatr Nephrol. 2012 Nov;27(11):2007-2016.

3. Westrope CA, Fleming S, Kapetanstrataki M, Parslow RC, Morris KP. Renal Replacement Therapy in the Critically Ill Child. Pediatr Crit Care Med. 2018 Mar;19(3):210-217.

4. Santiago MJ, López-Herce J, Urbano J, Solana MJ, del Castillo J, Ballestero Y, Botrán M, Bellón JM. Clinical course and mortality risk factors in critically ill children requiring continuous renal replacement therapy. Intensive Care Med. 2010 May;36(5):843-9.

5. Askenazi DJ, Goldstein SL, Koralkar R, Fortenberry J, Baum M, Hackbarth R, Blowey D, Bunchman TE, Brophy PD, Symons J, Chua A, Flores F, Somers MJ. Continuous renal replacement therapy for children ≤10 kg: a report from the prospective pediatric continuous renal replacement therapy registry. J Pediatr. 2013 Mar;162(3):587-592.

6. Jetton JG, Boohaker LJ, Sethi SK, Wazir S, Rohatgi S, Soranno DE, Chishti AS, Woroniecki R, Mammen C, Swanson JR, Sridhar S, Wong CS, Kupferman JC, Griffin RL, Askenazi DJ; Neonatal Kidney Collaborative (NKC). Incidence and outcomes of neonatal acute kidney injury (AWAKEN): a multicentre, multinational, observational cohort study. Lancet Child Adolesc Health. 2017 Nov;1(3):184-194.

7. Menon S, Broderick J, Munshi R, Dill L, DePaoli B, Fathallah-Shaykh S, Claes D, Goldstein SL, Askenazi DJ. Kidney Support in Children using an Ultrafiltration Device: A Multicenter, Retrospective Study. Clin J Am Soc Nephrol. 2019 Oct 7;14(10):1432-1440.

8a. Paediatric Intensive Care Audit Network Annual Report 2020 (published February 2021): Universities of Leeds and Leicester: https://www.picanet.org.uk/wp-content/uploads/sites/25/2021/02/PICANet2020_AnnualReportSummary_v1.0.pdf.

8b. Paediatric Intensive Care Audit Network Annual Report 2010 - 2012 (published September 2013): Universities of Leeds and Leicester. [Online] Available from http://www.picanet.org.uk/Audit/Annual-Reporting/PICANet Annual Report 2013 Summary.pdf

9. Westrope C, Morris K, Burford D, Morrison G. Continuous hemofiltration in the control of neonatal hyperammonemia: a 10-year experience. Pediatr Nephrol. 2010 Sep;25(9):1725-30.

10. Bojan M, Gioanni S, Vouhé PR, Journois D, Pouard P. Early initiation of peritoneal dialysis in neonates and infants with acute kidney injury following cardiac surgery is associated with a significant decrease in mortality. Kidney Int. 2012 Aug;82(4):474-81.

11. Diaa M. Prismaflex user's manual. http://www.scribdcom/doc/141000459/Prismaflex

12. Hothi DK, St George-Hyslop C, Geary D, Bohn D, Harvey E. Continuous renal replacement therapy (CRRT) in children using the AQUARIUS. Nephrol Dial Transplant. 2006 Aug;21(8):2296-300.

13. Sohn YB, Paik KH, Cho HY, Kim SJ, Park SW, Kim ES, Chang YS, Park WS, Choi YH, Jin DK. Continuous renal replacement therapy in neonates weighing less than 3 kg. Korean J Pediatr. 2012 Aug;55(8):286-92.

14. Hackbarth RM, Eding D, Gianoli Smith C, Koch A, Sanfilippo DJ, Bunchman TE. Zero balance ultrafiltration (Z-BUF) in blood-primed CRRT circuits achieves electrolyte and acid-base homeostasis prior to patient connection. Pediatr Nephrol. 2005 Sep;20(9):1328-33.

15. Ronco C, Garzotto F, Brendolan A, Zanella M, Bellettato M, Vedovato S, Chiarenza F, Ricci Z, Goldstein SL. Continuous renal replacement therapy in neonates and small infants: development and first-in-human use of a miniaturised machine (CARPEDIEM). Lancet. 2014 May 24;383(9931):1807-13.

16. Lorenzin A, Garzotto F, Alghisi A, Neri M, Galeano D, Aresu S, Pani A, Vidal E, Ricci Z, Murer L, Goldstein SL, Ronco C. CVVHD treatment with CARPEDIEM: small solute clearance at different blood and dialysate flows with three different surface area filter configurations. Pediatr Nephrol. 2016 Oct;31(10):1659-65.

17. Askenazi D, Ingram D, White S, Cramer M, Borasino S, Coghill C, Dill L, Tenney F, Feig D, Fathallah-Shaykh S. Smaller circuits for smaller patients: improving renal support therapy with Aquadex™. Pediatr Nephrol. 2016 May;31(5):853-60.

18. Coulthard MG, Sharp J. Haemodialysis and ultrafiltration in babies weighing under 1000 g. Arch Dis Child Fetal Neonatal Ed. 1995 Nov;73(3):F162-5.

19. Everdell NL, Coulthard MG, Crosier J, Keir MJ. A machine for haemodialysing very small infants. Pediatr Nephrol. 2005 May;20(5):636-43.

20. Hothi DK. Designing technology to meet the therapeutic demands of acute renal injury in neonates and small infants. Pediatr Nephrol. 2014 Oct;29(10):1869-71.

21. Laskin BL, Foster BJ. Extracorporeal therapy for the smallest children. Lancet. 2014 May 24;383(9931):1785-6.

22. Hussey MA, Hughes JP. Design and analysis of stepped wedge cluster randomized trials. Contemp Clin Trials. 2007 Feb;28(2):182-91.

23. Matthews JNS, Forbes AB. Stepped wedge designs: insights from a design of experiments perspective. Stat Med. 2017 Oct 30;36(24):3772-3790.

24. Brierley J, Larcher V. Compassionate and innovative treatments in children: a proposal for an ethical framework. Arch Dis Child. 2009 Sep;94(9):651-4.

25. University of Liverpool. Research without prior consent (deferred consent) in trials investigating the emergency treatment of critically ill children: CONNECT study guidance. 2015. https://www.liverpool.ac.uk/media/livacuk/iphs/Research,without,prior,consent,in,trials,investigating,the,emergency,treatment,of,critically,ill,children,CONNECT,guidance,July,2015.pdf

For peer review only

Figure 1: I-KID Study design

I-KID Study Step-wedge Cluster design

**Sequence**	**Period 1**	**Period 2**	**Period 3**	**Period 4**	**FU**
**S1**	**C**	**T** **I**			
**S2**	**C**		**T** **I**		
**S3**	**C**			**T** **I**	

S1, S2, S3 = sequences in SW design – two centres randomized to each sequence to switch to the intervention phase

C = Control Period - Conventional treatment in unit

T = Transition Period - Results from 1st patient not used for primary outcome

I = Intervention Period - whole unit uses NIDUS

FU = Follow up is up to one month after Last patient

Each of the data collection periods in each sequence (C and I) will be 4.5 months long.

Figure 1: I-KID study design sequence (The diagram is indicative. The 4 data collection periods are each 4.5 months. The transition period is up to 2 months and the follow up period for final recruits is one month).

Confidential: For Review Only

If 6 hr ultrafiltration (blue boxes) not reached, record all data under *Finish the Study-Period* heading of *Record sheet for UF* document when baby taken off, however short the UF time.

I-KID Study – Patient Timeline (Prismaflex, Aquarius, NIDUS)

Ref: I-KID Protocol 6.0
For detailed information always refer to full protocol.

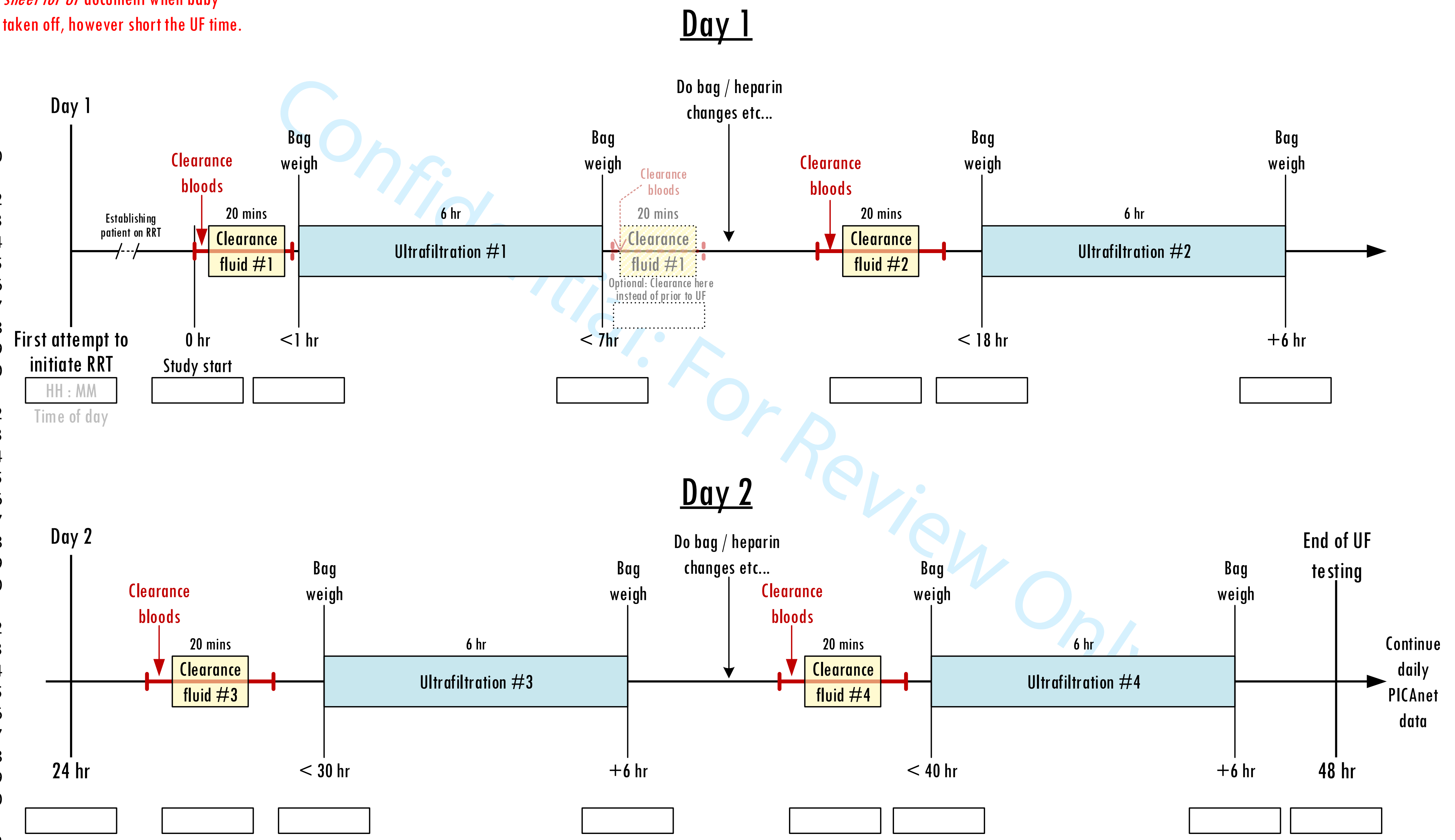

Confidential: For Review Only

7419 I-KID Patient Timeline (PrisAquNIDUS) v2.0 dated 23.09.2020.vsdx

PD ONLY

I-KiD Study – Patient Timeline (for Peritoneal Dialysis)

Ref: I-KID Protocol 5.0
For detailed information always refer full protocol.

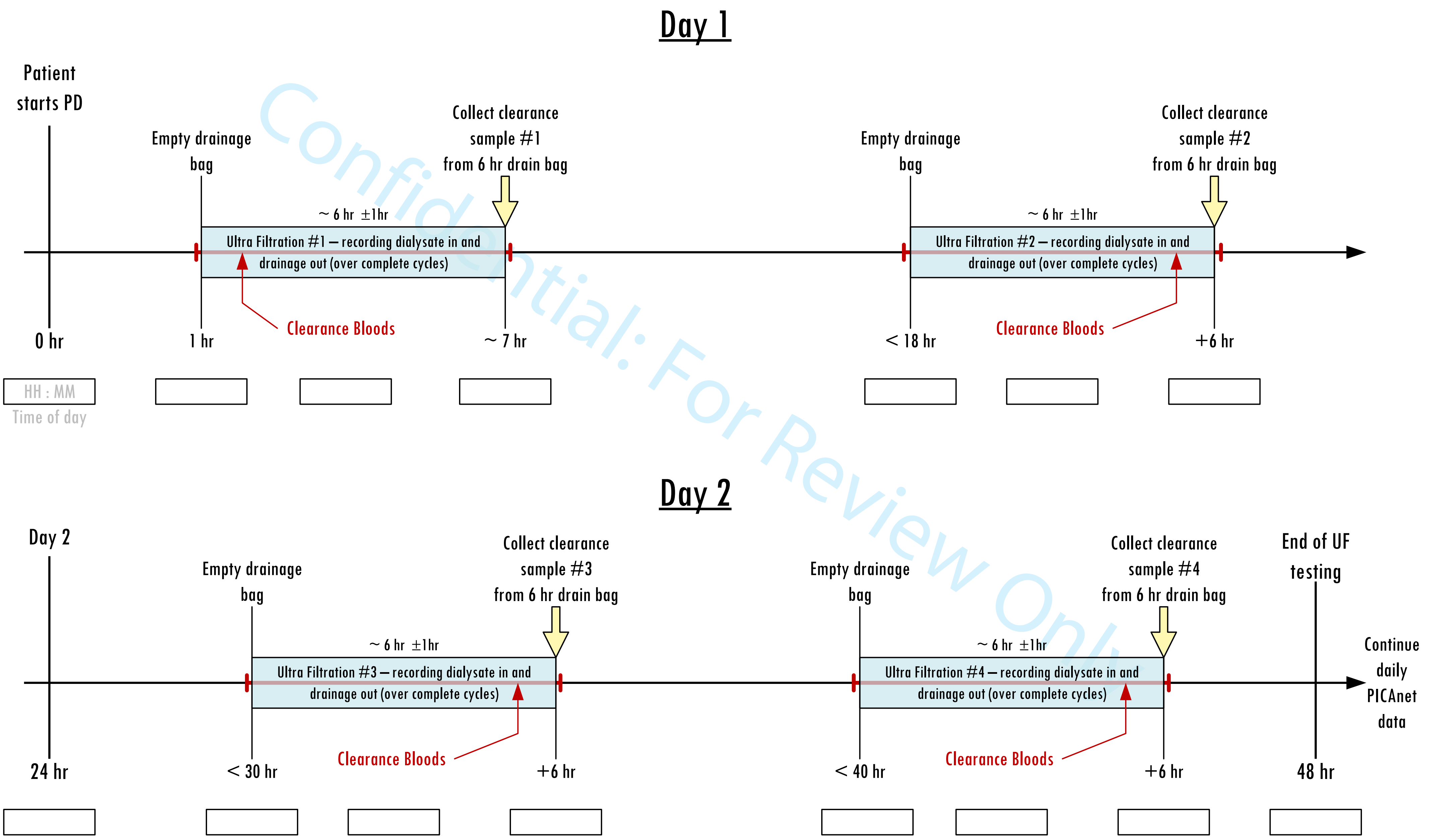

Confidential: For Review Only

I-KID Patient timeline (PD) – Version 1.0 (20181219)

Protocol	Amendments
V1.0 07/12/2015	**Original REC Submission**
V2.0 14/12/2017<br>Notice of no objection 16/02/2018	**Submitted to the MHRA as a Clinical Investigation**
V3.0 22/12/2017<br><br>Approved 21/02/2018	**Submitted to REC and HRA as a Clinical Investigation and included the following changes**<br>**1.** Nidus confirmed as a non-CE marked device<br>**2.** Submission changed to a clinical investigation not CTIMP<br>**3.** Changes to protocol including changes to consent process for two parents with conflicting views on consent<br>**4.** Changes to patient information sheet, for both the control and intervention arms and changes to formatting of consent form.<br>**5.** Addition of site: Evelina London Children's Hospital
V4.0 and V5.0 28/09/2018<br><br>Approved 31/10/2018	**Submitted to REC, HRA and MHRA and included the following changes. The MHRA requested updates to the changes therefore protocol version 4.0 was not approved. The changes resulted in the approved protocol V5.0**<br>Changes to Protocol and Patient information sheets to include the following:<br>**1.** Deferred consent in emergency situations<br>**2.** Inclusion of an estimated body weight in emergency situations<br>**3.** Process of using Nidus on compassionate grounds<br>**4.** Safety reporting<br>**5.** Update to patient information sheets to reflect the new data protection requirements
V6.0 15/02/2020<br><br>Approved 27/08/2020	**Submitted to REC, HRA and MHRA and included the following changes.**<br>**1.** Updates to protocol for clarification of the wording<br>**2.** PIS and Consent form documents for Bereaved Parents<br>**3.** Updates to documents from Nidus to NIDUS to match the trademark.

w Only
